# Performance optimization of black cotton soil stabilized with FGD gypsum and cement via response surface methodology

**DOI:** 10.1038/s41598-025-09159-9

**Published:** 2025-07-02

**Authors:** Chidananda M. Linganagoudar, G. Shiva Kumar, M. S. Ujwal, Varun S. Ullur, Poornachandra Pandit

**Affiliations:** 1 Department of Civil Engineering, Dayananda Sagar College of Engineering, Bengaluru & Visvesvaraya Technological University, Belagavi, India; 2 Department of Civil Engineering, Manipal Institute of Technology, Manipal Academy of Higher Education, Manipal, 576 104 India

**Keywords:** Black cotton soil, Cement, Flue gas desulfurization gypsum, Engineering properties, Response surface methodology, Civil engineering, Materials science

## Abstract

The growing demand for sustainable infrastructure solutions has driven the exploration of alternative materials for soil stabilization, especially for problematic soils such as black cotton (BC) soil. Owing to its high shrink-swell behavior, BC soil poses significant challenges in construction and pavement applications. This study evaluated the potential use of cement (up to 9.24%), flue gas desulfurization (FGD) gypsum (up to 3.41%), and industrial byproducts from thermal power plants as stabilizing agents to enhance the geotechnical properties of BC soil. A central composite design under the framework of response surface methodology (RSM) was employed to optimize the mix proportions and assess the effects on the unconfined compressive strength (UCS), California bearing ratio (CBR), and plasticity index (PI). The findings demonstrated substantial improvements in soil strength and a significant reduction in plasticity. The optimum mixture of 9.24% cement and 3.41% FGD gypsum yielded a desirability score of 71%, indicating an effective balance between strength gain and workability. This study underscores the viability of using FGD gypsum as a sustainable and eco-friendly soil stabilizer, offering an economical and efficient method for improving subgrade performance in flexible pavement systems. The results contribute to advancing green construction practices by utilizing industrial waste in geotechnical applications.

## Introduction

Black cotton (BC) soil, predominantly found in several regions of India, is an expansive clay characterized by high shrink-swell potential, low bearing capacity, and considerable volumetric changes in response to moisture variations. These geotechnical issues pose severe challenges in civil infrastructure development, especially in the construction of pavements, embankments, and foundations. The high montmorillonite content in BC soil results in cracking during dry seasons and heaving during wet seasons, often leading to structural distress and increased maintenance costs^1–3^. Owing to rapid urbanization and the limited availability of suitable land, construction on weak or problematic soils has become increasingly necessary. The scarcity of expansive soils presents challenges worldwide, making stabilizing black cotton (BC) soil to support sustainable infrastructure development crucial. In India, where 80% of transportation demand is met by road networks, stabilizing BC soil is of paramount importance for improving the quality of pavement^4–6^. The country’s National Highway network spans approximately 65,000 km, playing a major role in economic, industrial, and social advancement by linking production and consumption hubs. The need for well-designed highways increases with population growth, which emphasizes how crucial it is to stabilize BC soil. Predominantly found in Karnataka, Gujarat, Maharashtra, and Madhya Pradesh, which cover approximately 20% of India’s land, BC soil is rich in potash, iron, lime, alumina, and calcium and originates from the weathering of lava rocks on the Deccan and Malwa plateaus^7–9^. Despite its geographical importance, the subgrade quality and stability of BC soil are critical factors affecting pavement performance and longevity, necessitating effective stabilization techniques for sustainable infrastructure. Flexible pavements are frequently used because of their reduced construction costs; however, they are vulnerable to fatigue, rutting, and thermal cracking, with rutting being the most common problem^10–12^. The stability of the subgrade soil is essential for supporting traffic loads; however, BC soil, which comprises approximately 20% of India’s soil, is highly compressible and has a low bearing capacity. Soil stabilization techniques have long been employed to improve soil properties, with the first documented test conducted in 1904 in the United States. Flexible pavements constructed over expansive soils such as black cotton soil are prone to structural distress, including cracking, rutting, and heaving, primarily due to the high shrink-swell potential and low strength of the soil^1,13,14^. Surface rutting can also result from poor asphalt mix design, incorrect compaction, or the use of soft binders, even if subgrade deformation is a major contributing component^15–17^.

Despite extensive research on the resilient and permanent behaviour of granular materials, there has been limited focus on the permanent deformation of stabilized subgrade soils. Understanding the behaviour of stabilized subgrades is essential for designing durable and resilient flexible pavements, particularly in areas where the soil is prone to deformation and rutting. To guarantee the long-term functionality and sustainability of India’s road infrastructure, several issues must be resolved^18^. The stabilization of such problematic soils is a well-established technique to improve their engineering properties. Among the various stabilizers, ordinary Portland cement (OPC) is widely used because of its ability to increase strength, reduce plasticity, and bind soil particles effectively through hydration and pozzolanic reactions. However, there are sustainability issues because cement manufacturing uses a large amount of energy and produces high greenhouse gas emissions.

Black cotton (BC) soil presents considerable challenges in construction because of its expansive nature, driven by the presence of montmorillonite clay minerals, which cause high shrinkage and swelling with moisture fluctuations^19^. Predominantly found in semiarid regions, BC soil undergoes substantial volumetric modifications in all three dimensions as moisture levels vary, leading to a low bearing capacity when moist and pronounced cracking when dry. The high clay content and small interparticle gaps of soil cause it to retain too much water and have cohesive qualities^20^. While fertile, BC soil lacks key engineering qualities, namely, mechanical strength, permeability, and toughness, making it unfit for construction. Seasonal moisture variations exacerbate its instability, causing significant ground heave and structural cracking^18^,^21-23^. This instability poses particular challenges in pavement construction, as BC soil subgrades experience large volumetric shifts, compromising the integrity and durability of road structures^24^. Addressing these issues is essential for developing resilient infrastructure in regions dominated by BC soil.

To effectively manage waste materials and support the development of sustainable infrastructure, pavement construction demands creative solutions. This study examined the use of FGD gypsum and other industrial waste products as stabilizing agents for BC soil in pavement construction applications^25,26^. Coal-fired power plants are a key source of electricity worldwide, and flue gas desulfurization plants are essential for lowering the emission of sulfur dioxide (SO₂) and reducing its effects on the environment^27^. FGD gypsum (CaSO₄·2 H₂O), a byproduct of this process, is generated in vast quantities and shares many of the same physical and chemical characteristics as natural gypsum^28^. In contrast to natural gypsum, which has an 87.1% content, FGD gypsum is mainly composed of gypsum and quartz, with CaSO₄·2 H₂O percentages ranging from 84 to 99.6%. The specific density of gypsum is approximately 2.2 g/cm³, which is slightly less than that of natural gypsum (2.3 g/cm³)^29^. In contrast to natural gypsum, which contains 59% CaO and 41% SO₃, FGD gypsum contains 38% CaO and 54% SO₃^30^. Landfills and the agricultural industry have created large amounts of FGD gypsum. According to a 2019 report by the American Coal Ash Association (ACAA), the United States produced more than 23 million tonnes of FGD gypsum, with a 50% reuse rate. Similarly, the European Coal Combustion Products Association estimates that approximately 10 million tons were generated in Europe in 2016. FGD systems are now being installed for 60 GW of capacity by the National Thermal Power Corporation (NTPC), whereas the Central Electricity Authority (CEA) in India has planned these systems for 415 coal-fired boilers with a total capacity of 160 GW^31^. The production of 6–7 million tonnes of FGD gypsum per year is anticipated in India, making it a feasible material for high-volume uses such as pavement buildings^32^. Through the extra gypsum concept, FGD gypsum also offers a viable substitute for cement in soil–cement stabilization. This enhances the engineering qualities of soil‒cement combinations while lowering the amount of cement used. However, determining the ideal gypsum concentration is important since too much gypsum might cause supersaturated gypsum, which weakens the material^33^. In mixture design, FGD gypsum is essential when it is used with water and ordinary Portland cement (OPC)^34^. Black cotton (BC) soil stabilization for pavement construction is the main goal of this work, which attempts to determine the correlations between these factors for efficient soil–cement–FGD gypsum applications. A viable way to address these demands effectively and economically is through the use of this substitute material for soil stabilization^35^.

The dosage of the fly ash-based geopolymer was optimized for soil stabilization via response surface methodology (RSM). This study evaluated the California bearing ratio (CBR) to assess the performance of stabilized soil. With a face-centered central composite design (FCCCD), the fly ash-to-activator ratio (R) was varied between 0.6 and 2.0, and the curing duration was 0–28 days. The optimal dosage was *R* = 2.0 with a curing period of 27.98 days, resulting in a maximum CBR of 40.03%. The model showed high predictive strength, with an R² of 0.9613. The second study, which uses a similar experimental approach, targeted unconfined compressive strength (UCS) as the performance metric. The optimum fly ash-to-activator ratio (FAR) was 1.5, with 22.75 days of curing, yielding a maximum UCS of 1606.14 kPa. This model also demonstrated excellent accuracy (R² = 0.9884). While the first paper highlights the strength of pavement design, the second emphasizes structural stability. Both studies confirm that fly ash-based geopolymers are sustainable and effective alternatives to conventional stabilizers and that RSM is a powerful tool for dosage optimization with minimal testing^36,37^. With increasing urbanization and limited land availability, construction increasingly occurs on problematic soils such as black cotton (BC) soil. Predominantly found in parts of India, BC soil is characterized by high shrink-swell potential due to montmorillonite clay minerals, resulting in low strength and bearing capacity. These attributes challenge the durability of flexible pavements, where rutting and cracking are common. To address these issues, soil stabilization is essential. Cement is a well-known stabilizer; however, its production is resource intensive. Industrial byproducts such as FGD gypsum offer promising alternatives due to their pozzolanic nature and environmental benefits. FGD gypsum contains significant amounts of calcium and sulfur, facilitating strength gain through the formation of cementitious compounds. While fly ash and GGBS have been extensively studied, the use of FGD gypsum in soil stabilization remains underexplored. This study focuses on integrating cement and FGD gypsum, optimized via the RSM, to improve BC soil performance. This novel approach not only recycles industrial waste but also supports sustainable pavement construction.

### Research objectives and scope

The primary goals of this study were to determine the ideal ratios of cement to flue gas desulfurization (FGD) gypsum for stabilizing black cotton (BC) soil and to assess improvements in key geotechnical characteristics. The focus was placed on key parameters, including the unconfined compressive strength (UCS), California bearing ratio (CBR) under both soaked and unsoaked conditions, and plasticity index (PI), which were chosen for their critical importance in flexible pavement design as indicators of subgrade strength, load-bearing capacity, and soil shrink-swell potential. Predictive models for these parameters were developed via response surface methodology (RSM), providing a structured and efficient optimization framework. The accuracy and reliability of these models were confirmed through analysis of variance (ANOVA), ensuring a statistically sound representation of the experimental findings. The CBR test was chosen to assess the soil’s load-bearing capacity, which is closely linked to how well it performs as a subgrade material in pavement systems. To evaluate the structural performance of stabilized soil, UCS tests provide information on its shear strength and durability under axial loading circumstances. Atterberg limit tests, including measurements of the plasticity index, liquid limit, and plastic limit, were conducted to monitor changes in soil plasticity and workability factors that influence volumetric stability, shrink-swell behavior, and construction feasibility. Additionally, tests for swelling potential and compaction characteristics were carried out to gain further understanding of the soil’s volume change behavior and to fine-tune the mix design for practical implementation. Collectively, these tests establish a comprehensive evaluation framework that supports the development of an effective and sustainable soil stabilization strategy using cement and FGD gypsum.

## Materials and experimental methods

### Materials

#### Black cotton soil

The black colour, high concentration of montmorillonite clay, and typical traits of high expansiveness and difficult soils set BC soil, which comes from the Bidar district of the Uttar Karnataka region, apart. The characteristics of black cotton (BC) soil, which has a specific gravity of 2.50 and is categorized as high plastic clay (CH) by the Indian Standard soil classification, are displayed in Table 1. It exhibits notable expansiveness, with a high liquid limit of 74%, a plasticity index of 32%, and a free swell index of 38%. The soil has an optimum moisture content (OMC) of 21.6% and a modified Proctor maximum dry density (MDD) of 1.70 g/cc. Its unconfined compressive strength (UCS) is 107 kPa, whereas the California bearing ratio (CBR) is 4% (unsoaked) and less than 2% (soaked), suggesting poor subgrade strength. The permeability is low, at 0.22 × 10⁻⁷ cm/sec.

**Table 1:** Basic properties of black cotton soil.

Properties	Test method^38–45^	Average value	
Specific gravity G_s_	(IS 2720-Part 3/Section-1: 2016)	2.50	
Standard proctor compaction MDD (g/cc) OMC (%)	(IS 2720-Part 7: 2016)	1.62 22.0	
Modified proctor compaction MDD (g/cc) OMC (%)	(IS 2720-Part 8: 2015)	1.70 21.6	
Liquid limit (%)	(IS 2720-Part 5: 2015)	74	
Plastic limit (%)		41	
Plasticity index (%)		32	
Shrinkage limit (%)	(IS 2720-Part 6: 2016)	15	
Gravel (%)	(IS 2720-Part 4: 2015)	2	
Sand (%)		14	
Silt (%)		38	
Clay (%)		46	
Free swell index (%)	(IS 2720-Part 40: 2016)	38	
Indian standard soil classification		CH	
UCS (kPa) at modified proctor density	(IS 2720-Part 10: 2015)	107	
CBR % at standard proctor density	(IS 2720-Part 16: 2016)	Unsoaked	Soaked
		4	< 2
Coefficient of permeability (Cm/sec)	(IS 2720-Part 17:1986)	0.22 × 10^− 7^	

#### Cement

Ordinary Portland cement (OPC 43 grade) with a specific gravity of 3.15, a surface area of 2960 cm2/g, and qualities within the limitations was obtained from a local supplier for the experiments, which were conducted in accordance with IS 12,269 − 2013. The OPC 43-grade sample was dried at ≤ 105 °C to ensure moisture removal without impairing hydration.

#### Flue gas desulfurization gypsum

**Fig. 1:**
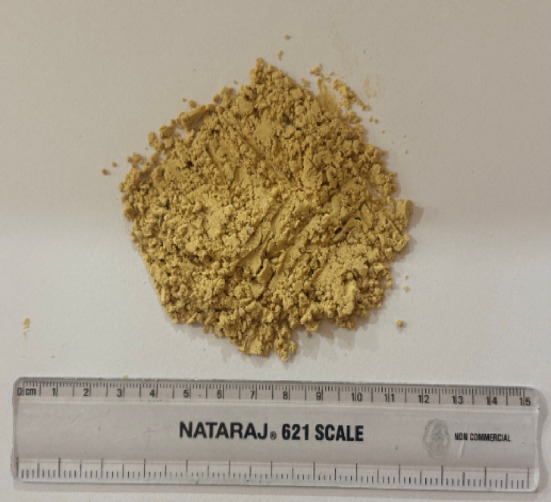
FGD gypsum.

**Fig. 2:**
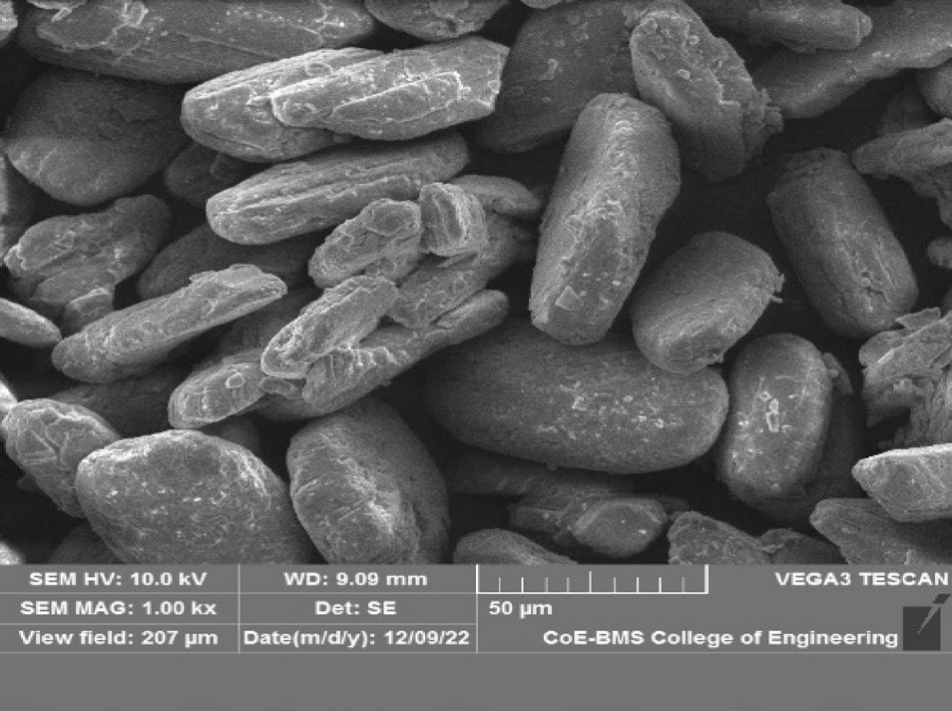
SEM image of FGD gypsum.

**Fig. 3:**
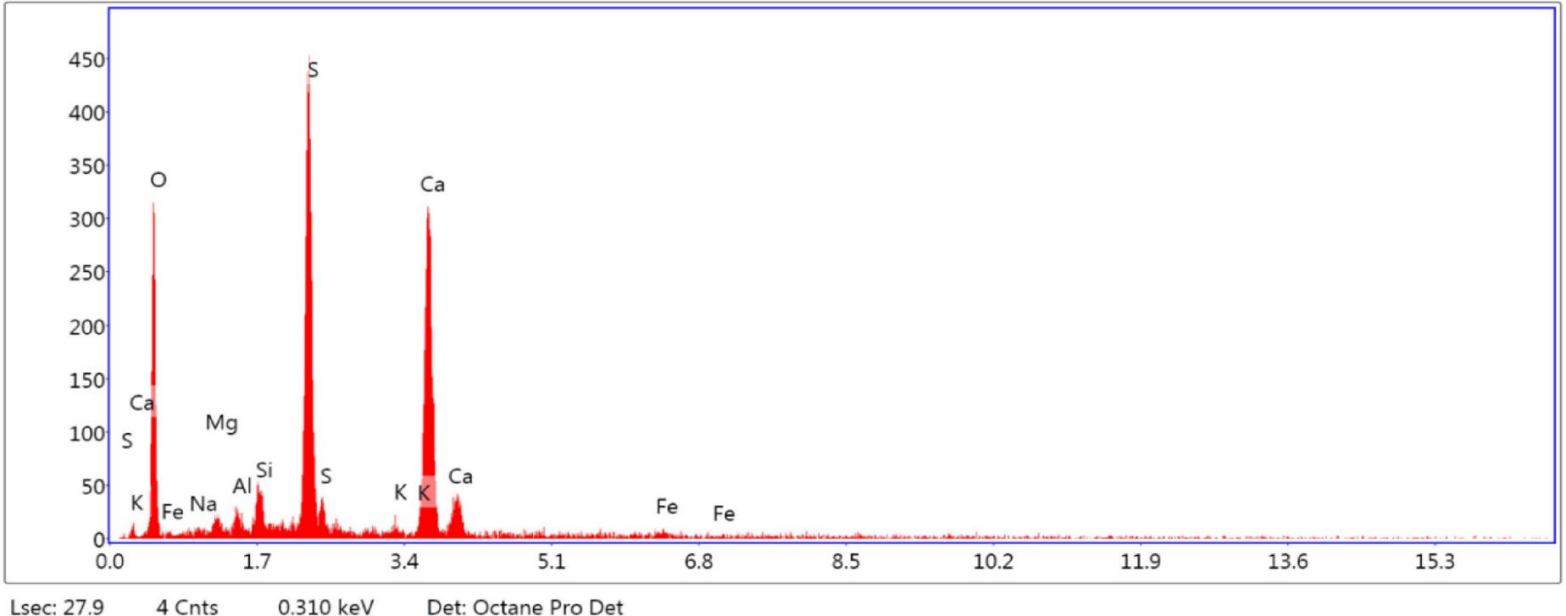
Chemical composition of FGD gypsum.

FGD gypsum from Fig. 1 was purchased from Udupi Power Corporation Limited (UPCL), a 1,200 MW coal-based thermal energy-producing facility with two 600 MW units situated in Karnataka, India. There are numerous important steps in the manufacturing of FGD gypsum. Calcium sulfite and calcium sulfate are first produced in a desulfurization unit when sulfur dioxide-containing flue gases combine with sorbents such as limestone or lime. Calcium sulfite is transformed into stable calcium sulfate (gypsum) by an oxidation step that is frequently assisted by air or oxygen. After that, specialized machinery such as fabric filters or mist eliminators removes gypsum from the flue gas. Finally, dewatering procedures eliminate surplus moisture, producing a fine powder with characteristics akin to those of natural gypsum. The FGD gypsum was oven-dried, and the lumps were removed before use. A microscopy image of FGD gypsum is shown in Fig. 2. The SEM micrograph captured at 1000× magnification reveals the surface morphology of the stabilized material, showing angular to subangular particles with elongated and platy structures. The rough texture and irregular edges of the particles indicate a crystalline nature, likely representing flue gas desulfurization (FGD) gypsum or cement hydration products such as calcium silicate hydrates (C-S-H) or ettringite. The particle sizes range between 20 μm and 80 μm, which is ideal for effective void filling and improved packing density in soil stabilization. The layered and flaky morphology enhances interparticle friction and bonding, contributing to the better shear strength and load-bearing capacity of the treated black cotton soil. Additionally, the visible voids and microcracks on the particle surfaces suggest moderate porosity, which can facilitate cementitious reactions and long-term strength gain. Overall, the SEM analysis confirmed the beneficial microstructural changes imparted by the addition of FGD gypsum and cement, supporting their effectiveness in improving the geotechnical behavior of expansive soils. Figure 3 shows the chemical composition of FGD gypsum, highlighting essential elements that contribute to its effective use in stabilizing black cotton soil. Prominent peaks were observed for calcium (Ca) at approximately 3.7 keV and for sulfur (S) at approximately 2.3 keV, with intensities reaching approximately 360 and 460 counts, respectively, confirming the significant incorporation of cement and flue gas desulfurization (FGD) gypsum, both of which are rich in these elements. Oxygen (O) showed a peak at ~ 0.52 keV with an intensity of approximately 100 counts, indicating the presence of oxides and silicate minerals. Silicon (Si), aluminum (Al), and magnesium (Mg) appeared in the energy range of 1.25–1.74 keV, with moderate intensities between 50 and 70 counts, reflecting the alumino-silicate structure characteristic of expansive clay minerals in BC soil. Iron (Fe) was detected at higher energy levels, approximately 6.4 to 7.0 keV, with a lower intensity of approximately 40 counts, indicating the presence of iron oxides. Minor peaks for potassium (K) and sodium (Na) were also observed at approximately 3.3 keV and 1.04 keV, respectively, suggesting contributions from feldspar and exchangeable cations. Table 2 shows the chemical composition of the FGD gypsum.

**Table 2:** Chemical composition of FGD gypsum.

Sl. No.	Chemical symbol	Percentage by weight (%)
1	CaO	31.25
2	SiO₂	2.80
3	Al₂O₃	0.75
4	Fe₂O₃	0.45
5	MgO	1.10
6	SO₃	45.50
7	Na₂O	0.35
8	K₂O	0.20
9	LOI	17.60

### Experimental methods using RSM

Developed by Box and Wilson, response surface methodology (RSM) is a potent empirical technique for analysing the connections between responses and controllable factors, particularly in situations when theoretical models are complicated or unavailable. Sequential experimentation is supported, variability is estimated, model adequacy is checked, reactions in uncharted territories are predicted, and knowledge is generated within the experimental domain. The RSM improves efficiency by saving time and cost, detecting outliers, and aiding decision-making under uncertainty. Beyond model fitting, it is fundamental in industrial experimentation and aligns with the philosophy of sequential learning. Software such as Minitab and Design Expert are commonly used for RSM analysis. While a global linear model can be used initially, second-order or higher polynomial models are usually needed to capture the curvature in factor interactions. The global linear equation, as represented in Eq. (1), can be used to formulate the RSM model. However, because interactions between input and output variables often exhibit curvature, a purely linear relationship is generally inadequate for representing these interactions. In such cases, second-order or higher-order polynomial models are more suitable.

1 $$R = ~\pi 0 + ~\pi _{1} A_{1} + \pi _{2} A_{2} + ...\pi _{n} A_{n} + \varphi$$

2 $$R = \pi _{0} + \mathop \sum \limits_{{a = 0}}^{n} \pi _{a} A_{a} + \mathop \sum \limits_{{a = 1}}^{n} \pi _{{aa}} A^{2} _{a} + \mathop \sum \limits_{{a < }}^{n} \sum \pi _{{ab}} A_{i} A_{j} + \varphi$$

Equation (1) describes a higher-order polynomial relationship between the input variables and the corresponding responses. Here, π₁ = π₂ = πₙ = 0, where π₁ and π₂ represent the coefficients associated with the first and second input variables, respectively. Similarly, A₁ and A₂ denote the first and second input variables, respectively, whereas Ψ accounts for the model’s error term.

In this study, RSM was employed to optimize the proportions of cement and flue gas desulfurization (FGD) gypsum for stabilizing black cotton soil. Design Expert software (version 21) was used to carry out the analysis via central composite design (CCD), which enabled the development of predictive models and the assessment of parameter significance. A total of thirteen experimental mixes were generated by varying the input levels of the cement and FGD gypsum, with key responses such as UCS, CBR, and PI measured to evaluate the soil performance. The RSM approach has proven beneficial in minimizing experimental trials while accurately identifying optimal mix proportions for improved geotechnical properties. As shown in Table 3, the combination of cement (C) and flue gas desulfurization gypsum (FGD) was optimized and modelled to improve the soil properties. On the basis of the experimental mix design derived from the RSM, a total of thirteen mixes were developed by varying the input parameters and their levels. The compositions of these mixtures, along with the test results, are given in Table 4.

**Table 3:** Factors (Variables) and levels.

Factors	Name	Unit	Levels	
			Low	High
A	Cement	%	0.76	9.24
B	FGD	%	0.59	3.41

### Experimental methods

The Atterberg limits of BC soil combined with cement and flue gas desulfurization gypsum in different proportions in accordance with the Indian Standard (IS:2720-Part 5–2015) were ascertained experimentally via the Casagrande apparatus and the thread rolling method (Fig. 2). The IS heavy compaction method was used to examine the densification parameters as well as the UCS and CBR. The goal of the IS heavy compaction test (IS:2720-Part 7-2016) was to determine the optimal water content and maximum dry density by densifying the soil samples in five layers using a 4.89 kg rammer that was dropped from a height of 450 mm, with 25 hammers blowing each layer. Through a CBR test (IS:2720-Part 16-2016), the strength of the BC soil-waste blends was evaluated by analysing their resistance to penetration under both damp and dry conditions at the ideal water content. The curing process for the soaked CBR samples was as follows: compressed samples were kept at room temperature (25 ± 2 °C) for 0, 7, 14, and 28 days to maintain a constant water content. Before testing, the samples were submerged in water for 96 h following curing.

For samples that were 75 mm and 38 mm high, the UCS test (IS:2720 Part 10–2015) was used to assess the strength improvement caused by adding industrial waste and performing axial compression until failure. Cylindrical BC soil samples that were 75 mm tall and 38 mm wide, densified to OMC, and completely cured were used for endurance tests. For curing times of 0, 7, 14, and 28 days at room temperature (25 ± 2 °C) for varying quantities of BC soil, BC soil + 6% C, and BC soil + 6% C + 4% FGD, the stabilized and untreated soil samples, respectively, were kept in a desiccator to maintain a constant water level. Parameters for fatigue test loading: Every sample was exposed to loads that were proportional to its UCS value, which was determined at the dosage levels that worked best. The treated and untreated soil samples were tested under cyclic loading at one-third, one-half, and two-thirds of their UCS values both before and after 28 days of curing. Cylindrical samples were made via the fatigue testing technique and placed on a testing frame with LVDTs integrated for tracking deformation. The load cell was carefully positioned in contact with the sample surface and calibrated. 3 shows the equipment and specimen arrangement. By employing specialized software within the control unit, specific parameters such as frequency, loading stress and waveform were set for the loading system. Both the data collection system and loading system were subsequently calibrated to initiate repeated loading on the sample. This procedure continued until failure was observed, with consistent monitoring of the vertical displacement during each cycle of loading via a data collection system that recorded the results in a data file. In addition to these acquired data, visual observations of the failure patterns of the samples offered additional observations. Microstructural studies were performed on BC soil samples treated with ideal contents of cement and FGD gypsum to analyse particle interlocking, pore size distribution, and soil matrix cohesion.

**Table 4:** Test results for different proportions of cement and FGD gypsum in the soil.

C	FGD	LL (%)	PL (%)	PI (%)	SL (%)	MDD (g/cc)	OMC (%)	k (cm/s)	UCS	CBR S	CBR US
5.00	2.00	41.4	28.2	13.2	7.4	1.98	16.14	1.33E-07	585	33.9	46.5
5.00	3.41	41.4	28.2	13.2	7.4	1.98	16.14	1.33E-07	585	33.9	46.5
2.00	1.00	39.2	28.3	10.9	7.1	1.92	19.06	3.11E-07	560	32.1	43
5.00	2.00	41.4	28.2	13.2	7.4	1.98	16.14	1.33E-07	585	33.9	46.5
0.76	2.00	52.3	28.9	23.3	8.9	1.91	17.56	2.05E-06	550	18.9	27.8
9.24	2.00	46.7	29.7	17	8.2	1.78	19.09	2.75E-07	528	31.5	40.5
5.00	0.59	52.1	30.4	21.62	8.2	1.84	18.8	2.21E-06	520	16.8	26
5.00	2.00	41.4	28.2	13.2	7.4	1.98	16.14	1.33E-07	585	33.9	46.5
8.00	1.00	41.4	28.2	13.2	7.4	1.98	16.14	1.33E-07	585	33.9	46.5
5.00	2.00	56.5	32.4	24.12	9.5	1.72	20.51	2.41E-06	488	16.1	21.1
2.00	3.00	31.2	19.2	12	7.6	1.85	18.49	1.98E-05	566	24.8	36.5
8.00	3.00	29.2	17.4	11.8	7	1.81	18.89	2.82E-05	538	24.1	35
5.00	2.00	33.2	19.7	13.5	8	1.75	19.69	2.21E-05	500	23.3	34.4

### Methodology

**Fig. 4:**
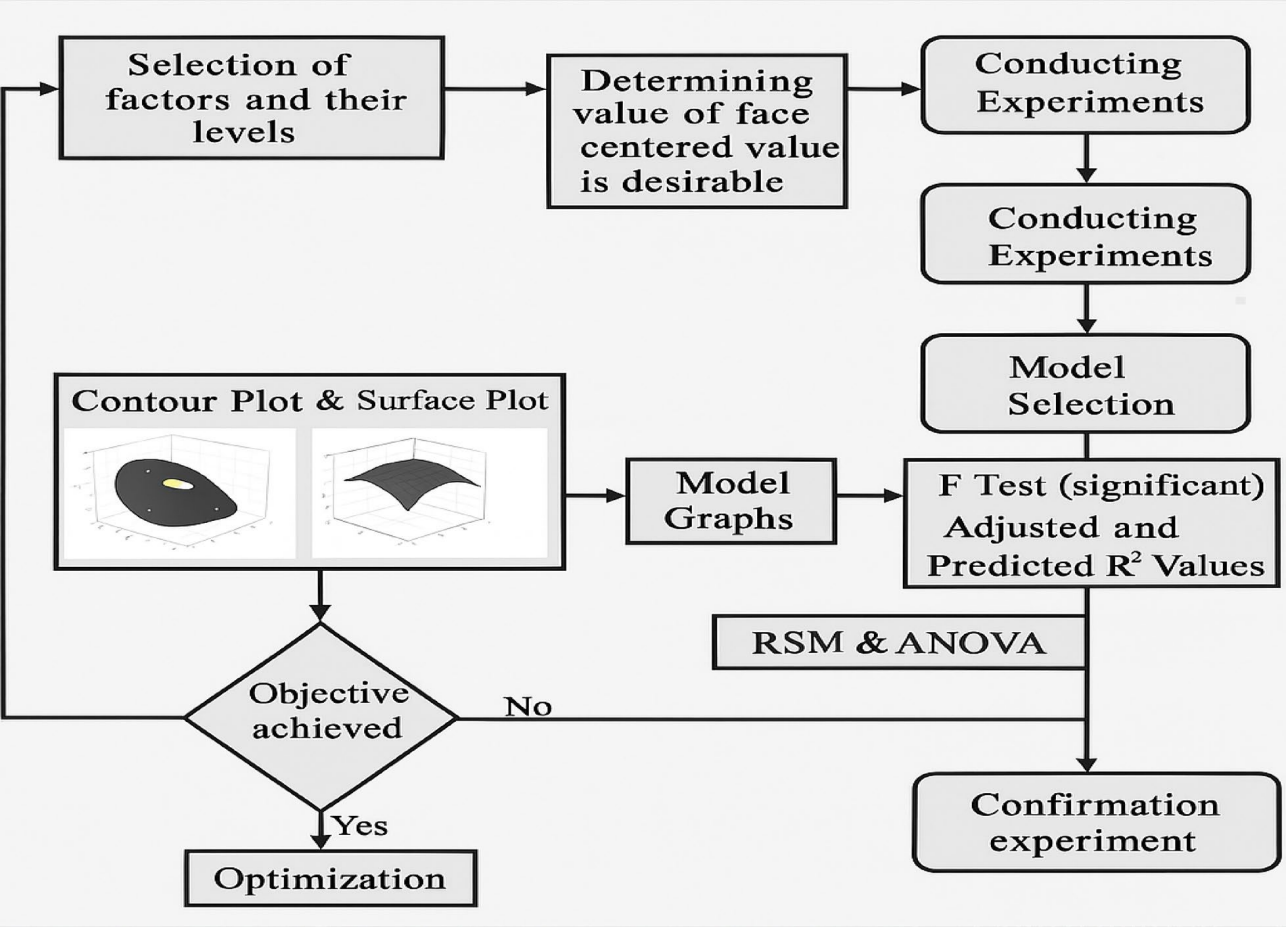
Methodology.

The flowchart (Fig. 4) provides a detailed representation of the response surface methodology (RSM) process, which is widely used for experimental design, optimization, and modelling. It consists of three main phases, namely, the initial phase, analysis, and decision, each guiding the researcher through a structured approach to studying the relationships between input factors and response variables. In the initial phase, the process begins with the selection of factors and their levels, where key independent variables that influence the response are identified and assigned specific values. Next, the face-centred design (FCD) is determined to ensure balanced experimental conditions, followed by selecting the response variables to be analysed. Once these parameters are set, the experiments are conducted according to a predefined design matrix, allowing data collection. After the experimental results are obtained, an appropriate mathematical model is selected on the basis of statistical considerations and the complexity of interactions between variables. The analysis phase involves processing the collected data to derive meaningful insights. Contour and surface plots are generated to visualize the interaction between variables and the response. Model graphs further aid in interpreting trends and patterns. The statistical significance of the model is assessed via an F test, which determines whether the model adequately explains the variation in the response. Additionally, adjusted and predicted R² values are computed to evaluate the model’s accuracy and predictive capabilities. Further validation is carried out through analysis of variance (ANOVA), which determines the significance of factors and their interactions. This phase ensures that the model is both statistically valid and practically useful for predicting responses under different conditions. In the decision phase, the results are examined to determine whether the experimental objective has been achieved. If the target criteria are not met, the process loops back to the analysis phase for further refinement, requiring adjustments to the model or additional experiments. If the objective is successfully met, optimization techniques are applied to fine-tune the input parameters for the best possible response. Finally, a confirmation experiment is conducted to validate the optimized model, ensuring that the predicted results align with the experimental outcomes. If the validation is successful, the model is considered reliable for practical applications.

## Results and discussion

### Atterberg limits

#### Liquid limit

**Fig. 5:**
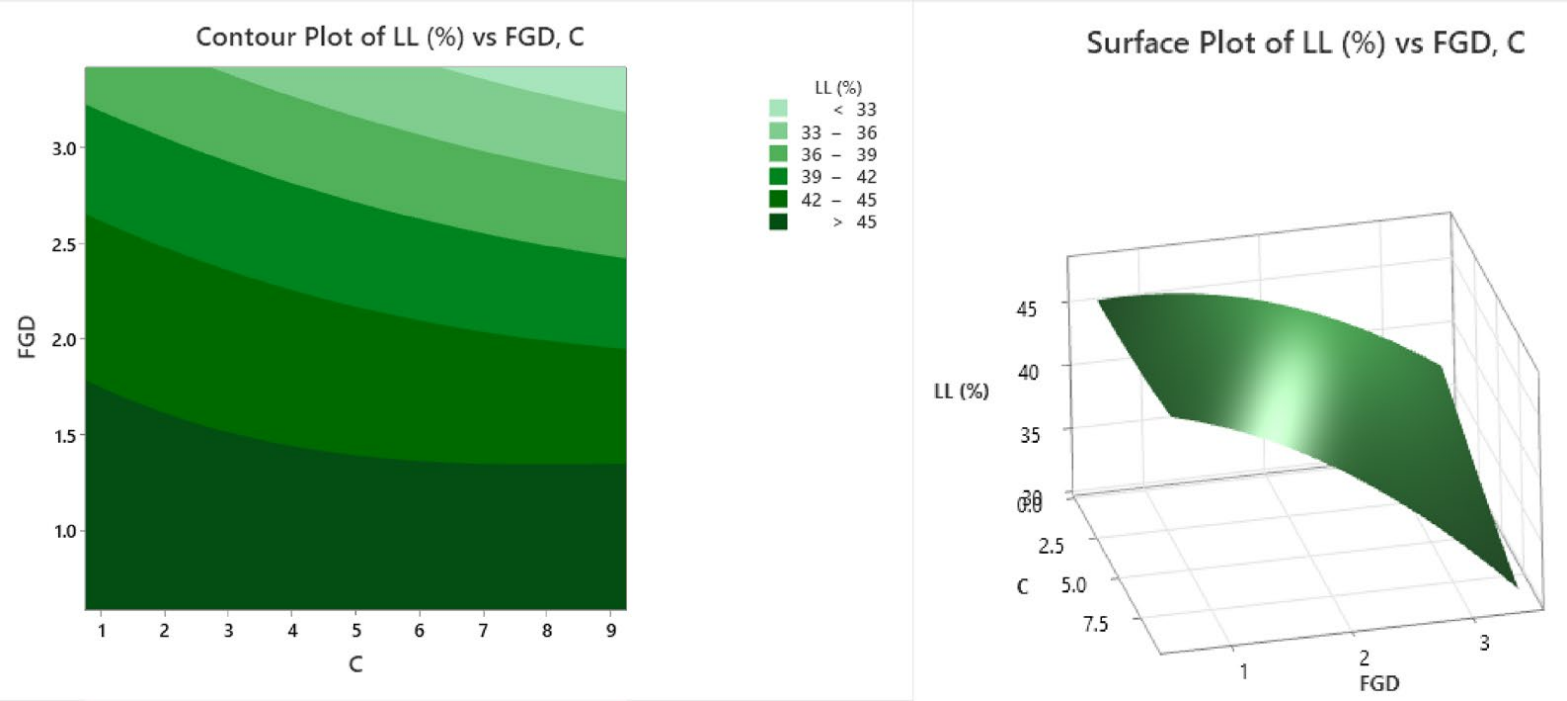
Counter plot and surface plot of LL.

The contour and surface plots (Fig. 5) represent the variation in the liquid limit (LL%) with respect to the FGD (fly ash‒gypsum dust) and cement content (C). The contour plot is a two-dimensional graphical representation that uses varying shades of green to depict LL% ranges. The lightest shade represents LL% less than 33%, followed by subsequent shading, representing 33–36%, 36–39%, 39–42%, 42–45%, and the darkest shade, representing LL% greater than 45%. The contour plot reveals that at lower FGD levels (approximately 0.75–1.5) and higher cement contents (approximately 6–9), the LL% values are highest, as shown in the darkest green zone, indicating values above 45%. As the FGD content increases (above 2.5) and the cement content decreases (below 3), the LL% values decrease, shifting into lighter green bands with LL% values falling below 36%. The surface plot provides a three-dimensional perspective of the same data, with the Z-axis representing LL% and the X- and Y-axes denoting FGD and C, respectively. The surface reaches its peak LL% value (approximately 45%) when the cement content is high and the FGD is low. As FGD increases and the cement content decreases, the LL% gradually decreases, forming a downwards slope in the surface plot. This trend mirrors that observed in the contour plot. These observations highlight that cement addition enhances the soil’s plasticity and water retention, thereby increasing the LL%, whereas excessive FGD reduces it due to its nonplastic, absorptive nature. From an engineering perspective, this trend underscores the need for an optimized mix design that balances cement, and the FGD content is critical for tailoring soil properties in applications such as subgrade stabilization, where specific LL ranges may influence the workability and compaction behaviour.

#### Plastic limit

**Fig. 6:**
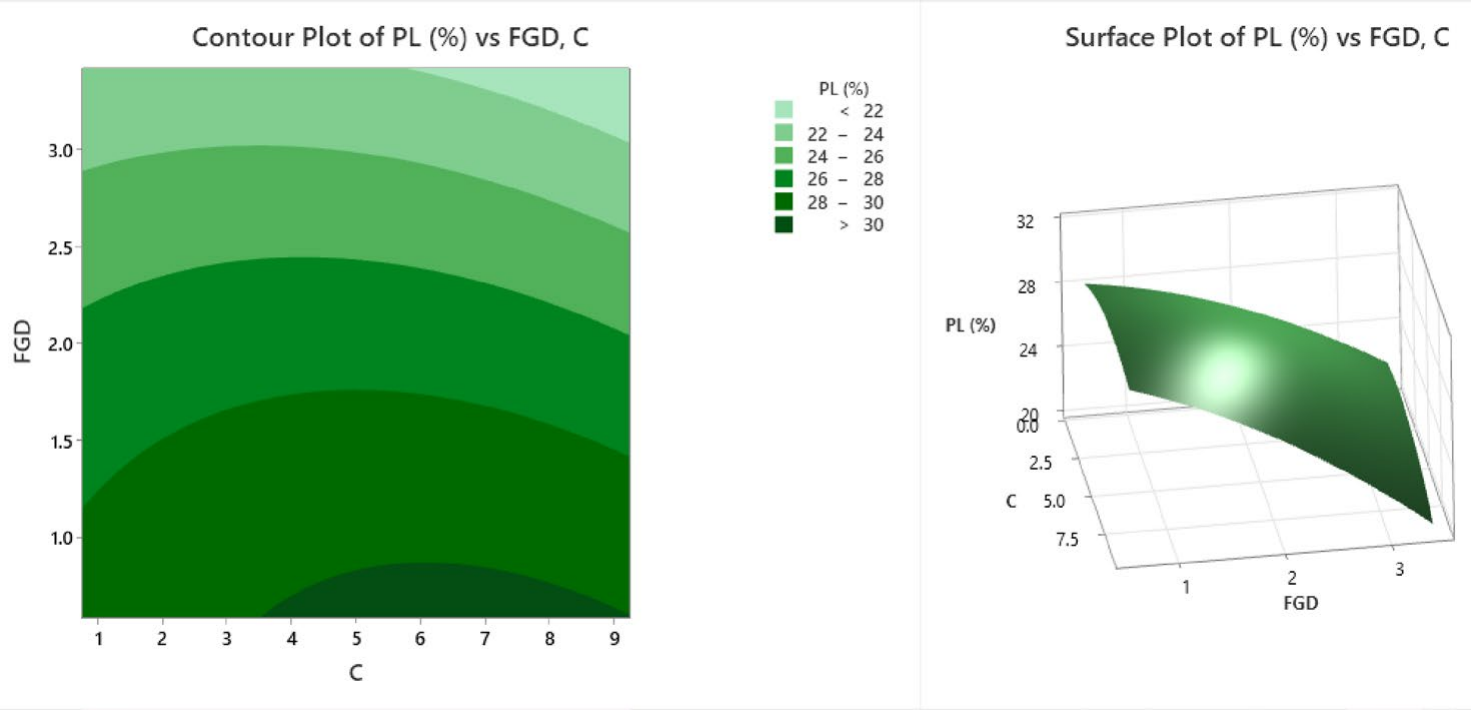
Counter plot and surface plot of the PL.

The provided contour and surface plots (Fig. 6) illustrate the variation in the plastic limit (PL%) in relation to two variables: FGD (fly ash-gypsum dust) and cement content (C). The contour plot displays a two-dimensional representation where different shades of green indicate specific PL% ranges. The lightest green color represents PL% less than 22, followed by progressively darker shading, representing ranges of 22–24%, 24–26%, 26–28%, and 28–30%, and the darkest green color indicates PL% greater than 30. From the contour plot, it is evident that a higher cement content (approximately 6–9) combined with a lower FGD content (approximately 0.75–1.5) results in the highest plastic limit values, falling in the dark green band (> 30%). Conversely, as the FGD increases (above 2.5) or the cement content decreases (below 3), the PL% decreases and falls into lighter green bands, indicating less desirable plastic behavior. This trend is further supported by the surface plot, which offers a three-dimensional view of the same relationship. In the surface plot, the Z-axis represents PL%, whereas the X- and Y-axes represent FGD and C, respectively. The surface peaks where FGD is low and the cement content is high confirm that this combination yields the highest PL values (approximately 32%). The surface gradually slopes down toward lower PL values as the FGD increases and the cement content decreases. This visual analysis emphasized the significant influence of both variables on soil plasticity. A higher cement dosage enhances the plasticity and workability of the soil, whereas an excessive FGD content tends to reduce the plastic limit because of its fine, nonplastic particles. This behavior reflects the beneficial role of cement in enhancing soil plasticity and workability, whereas excessive FGD composed of nonplastic, fine particles dilutes this effect. From a practical standpoint, these findings suggest that maintaining a high cement content with minimal FGD is crucial for applications requiring improved plasticity, such as subgrade improvement or slope stabilization.

#### Plasticity index

**Fig. 7:**
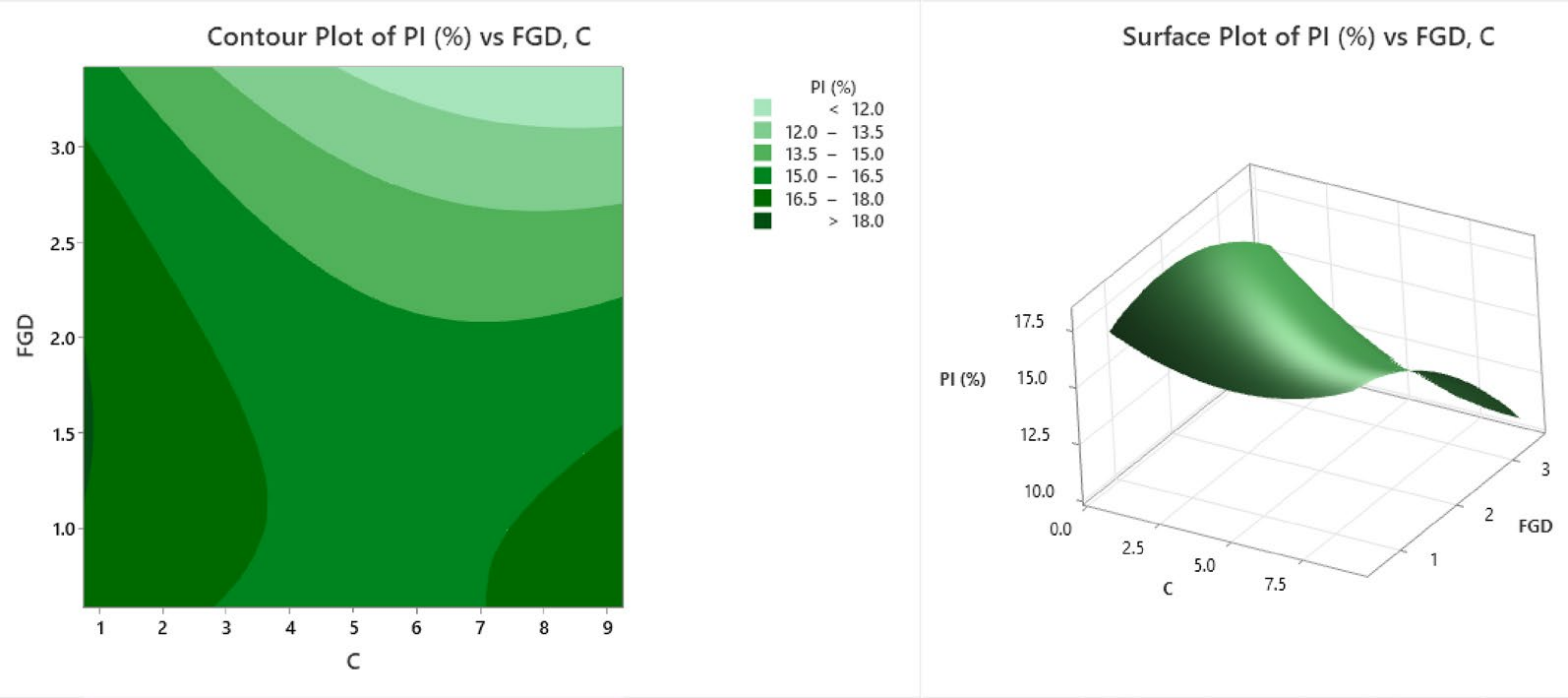
Counter plot and surface plot of the PI.

The contour and surface plots (Fig. 7) illustrate the influence of flue gas desulfurization (FGD) gypsum and cement content (C) on the plasticity index (PI%) of soil, a key indicator of soil plasticity and its potential for shrink-swell behaviour. In the contour plot, the variation in PI (%) is represented through a gradient of green shades. The lightest green area, representing PI values below 12%, is observed at high FGD levels (above 2.5%) and moderate-to-high cement contents (~ 6–9%), suggesting that this combination significantly reduces the plastic behaviour of the soil. As we move toward darker green zones, the PI values increase through the ranges of 12.0–13.5%, 13.5–15.0%, 15.0–16.5%, and 16.5–18.0%, with the darkest green areas indicating PI values above 18%. These higher PI zones occur predominantly at lower FGD levels (below 1.5%) and lower cement contents (< 4%), reflecting less effective stabilization. The surface plot provides a three-dimensional view of this relationship, showing a clear reduction in the PI (%) with increasing FGD content, especially above the 2% mark. The PI values reach their maximum (~ 18%) when both the FGD and cement contents are low, highlighting the high plasticity of the soil under minimal treatment. In contrast, the lowest PI values (~ 11%) occur at higher FGD and cement levels, emphasizing the importance of both additives in reducing plasticity. These plots reinforce that both FGD gypsum and cement contribute significantly to lowering the plasticity index of expansive soils. The results underscore the importance of incorporating adequate FGD and cement to mitigate the shrink-swell behavior of expansive soils. Such mix designs enhance soil stability, making them more suitable for applications such as road subgrades, foundations, and embankments in moisture-variable environments.

#### Shrinkage limit

**Fig. 8:**
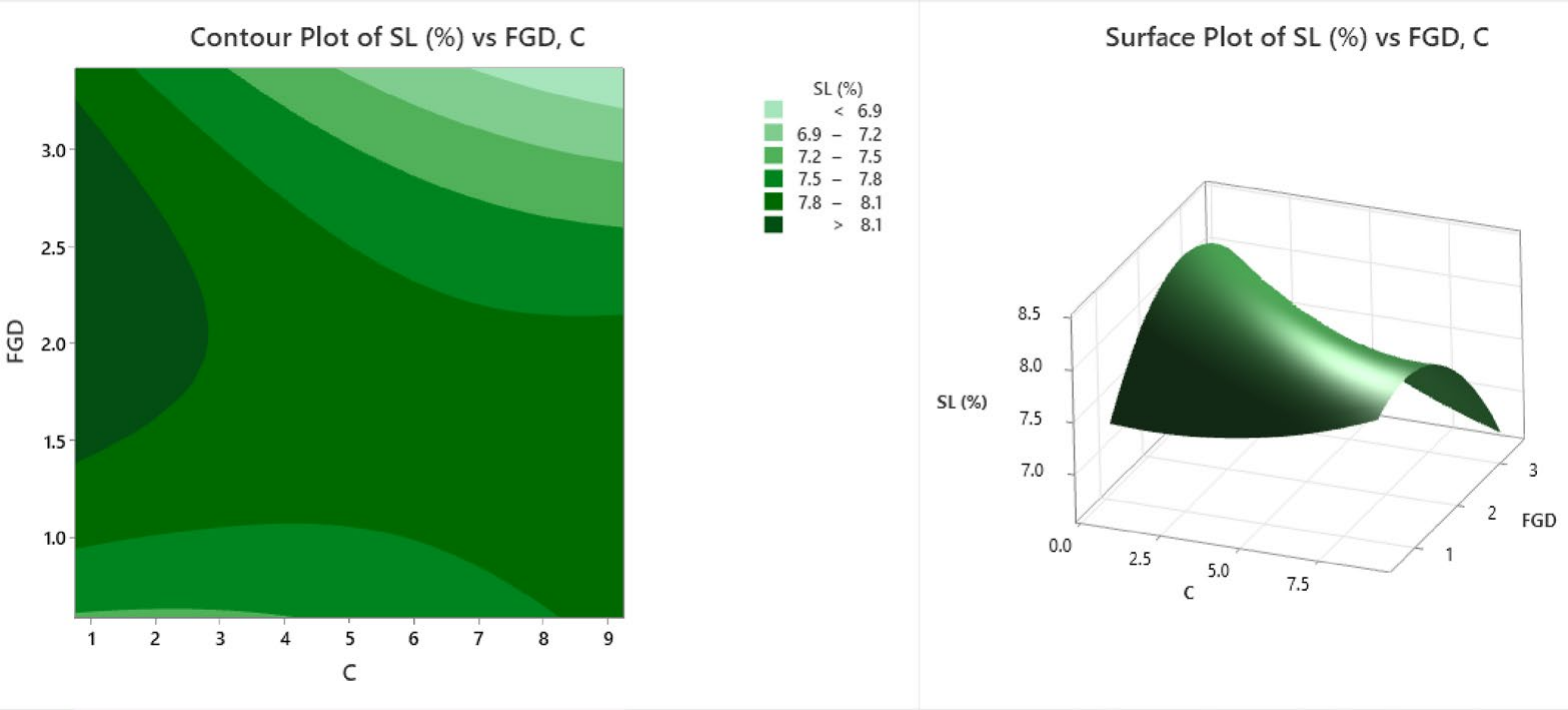
Counter plot and surface plot of SL.

The contour and surface plots (Fig. 8) illustrate the impacts of flue gas desulfurization (FGD) gypsum and the cement content (C) on the swelling limit (SL%) of the stabilized soil. The swelling limit is a critical parameter in assessing the change in soil volume when exposed to moisture and is particularly important in expansive soils. In the contour plot, different shades of green represent increasing SL (%) values across the FGD and cement composition spectra. The lightest green band indicates regions with the lowest swelling limit (< 6.9%), generally occurring at high FGD (> 2.5%) and moderate cement levels (~ 6–9%), suggesting that this combination effectively reduces expansive behaviour. The SL values increase through the following bands: 6.9–7.2%, 7.2–7.5%, 7.5–7.8%, and 7.8–8.1%, with the darkest green zones reflecting SL values greater than 8.1%. These highest SL values are concentrated in areas with low cement content (< 3%) and low-to-moderate FGD, indicating insufficient stabilization to control swelling. The surface plot provides a 3D visualization of the same relationship, showing a clear peak in SL (%) around moderate FGD (1.5–2%) and low cement content (~ 2–3%), where the swelling limit reaches above 8.5%. In contrast, the lowest SL values (~ 6.8%) are observed at higher cement dosages (~ 8–9%) and moderate-to-high FGD levels, suggesting that the expansive nature of the soil is well controlled when both stabilizers are effectively used. These visualizations demonstrate that the proper balance of cement and FGD significantly influences the swelling characteristics of treated soil. A relatively high cement content combined with adequate FGD gypsum can substantially reduce the swelling potential, increasing the suitability of the soil for construction. Conversely, inadequate cement content leads to insufficient binding, resulting in elevated SL values. These results highlight that a higher cement content, complemented by sufficient FGD, effectively limits swelling by enhancing particle bonding and reducing moisture sensitivity. For field applications, such as foundation support or pavement subgrades in expansive soil regions, the plots aid in identifying optimal mix proportions that ensure volumetric stability and long-term durability.

### Compaction characteristics

#### Maximum dry density

**Fig. 9:**
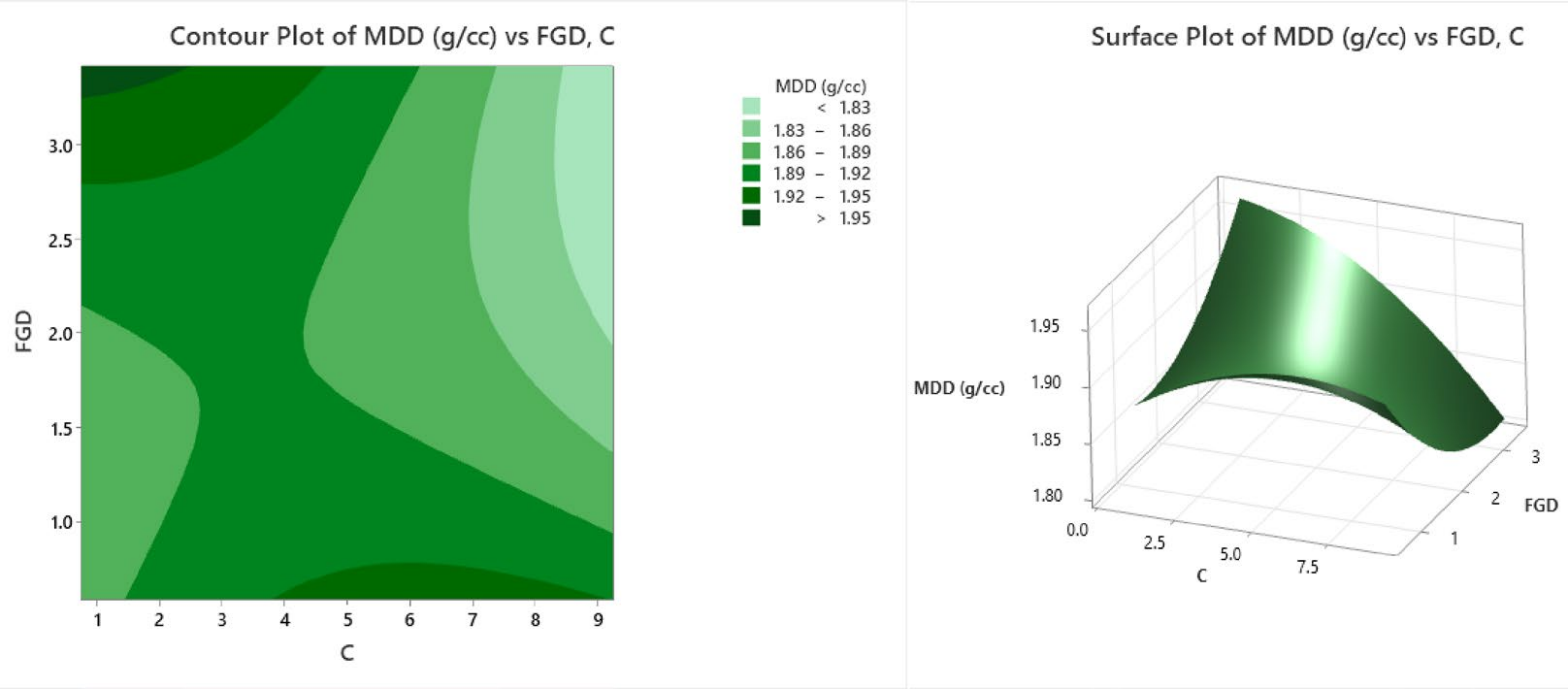
Counter plot and surface plot of the MDD.

The contour and surface plots (Fig. 9) depict the influence of flue gas desulfurization (FGD) gypsum and cement content (C) on the maximum dry density (MDD) of stabilized soil, a crucial parameter in evaluating the compaction characteristics of soil mixtures. In the contour plot, different shades of green represent varying ranges of the MDD in g/cc. The lightest green areas correspond to values less than 1.83 g/cc, indicating lower compaction densities, which are mostly located in regions with low cement and moderate FGD contents. As the color intensifies, the MDD values increase, ranging from 1.83 to 1.86 g/cc, 1.86–1.89 g/cc, 1.89–1.92 g/cc, and 1.92–1.95 g/cc. The darkest green zones represent areas where the MDD exceeds 1.95 g/cc, which is observed in the region of moderate FGD (approximately 2%) and moderate cement content (approximately 4–6%), indicating the most compacted and densified soil mix. The surface plot offers a 3D representation, emphasizing the interaction between FGD and cement in influencing the MDD. The plot features a distinct peak in the center region where both FGD and cement are at moderate levels, confirming that this combination yields the highest dry density, close to or above 1.95 g/cc. Toward the edges—particularly at lower cement contents (< 3%) and higher FGD levels (> 2.5%), the MDD values decrease to approximately 1.80–1.85 g/cc, as indicated by the sloping surface. This decrease suggests reduced soil compaction, likely due to excessive FGD, which may lead to poor particle packing or increased voids. A central peak confirmed the highest MDD at balanced additive levels. Toward the peripheries, especially at low cement and high FGD, the MDD decreases, indicating less effective compaction. From an engineering perspective, these results emphasize the importance of proportioning FGD and cement to achieve denser, more stable soil structures. The optimal MDD improves the load-bearing capacity and minimizes settlement risk, making such mixtures ideal for applications such as pavement subbases, embankments, and structural backfills.

#### Optimum moisture content

**Fig. 10:**
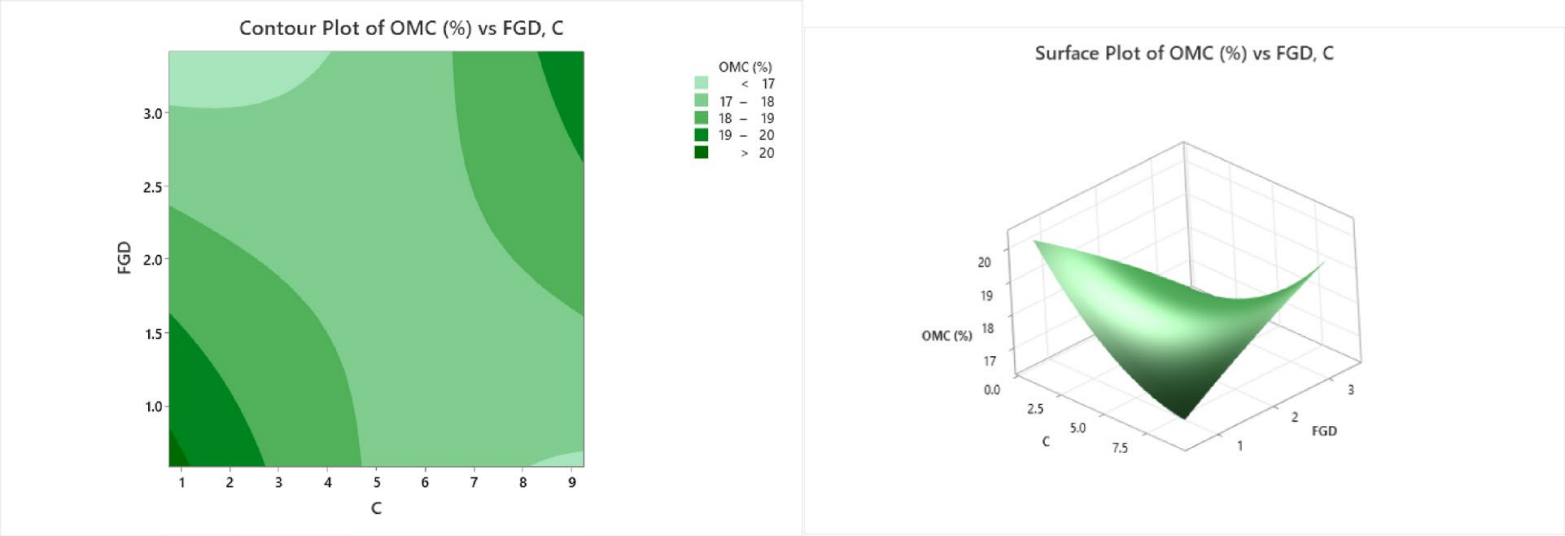
Counter plot and surface plot of OMC.

The contour and surface plots (Fig. 10) illustrate the variation in the optimal moisture content (OMC) with respect to flue gas desulfurization (FGD) gypsum and the cement content (C) during soil stabilization. The contour plot shows a two-dimensional view where different shades of green represent various OMC percentage bands. The lightest green areas indicate regions with OMC values less than 17%, which are predominantly found around moderate cement (approximately 5%), and lower FGD values (approximately 1.5%). As the color intensifies through green shading, the OMC ranges increase incrementally: 17–18%, 18–19%, 19–20%, and greater than 20%. The darkest green area, which is located towards the upper right of the plot, signifies high OMC (> 20%) associated with high levels of both FGD and cement. This implies that increasing both stabilizers simultaneously leads to greater moisture demand in the soil mixture. The surface plot provides a three-dimensional perspective of this interaction, clearly depicting a bowl-shaped curve with a minimum OMC observed at the middle range of both variables—approximately 1.5% FGD and 5% cement—where the OMC decreases to nearly 16.5–17%. As either the FGD or the cement content increases beyond this optimal point, the OMC increases sharply, exceeding 20% in the extreme corners of the surface. This behavior can be attributed to the water-absorbing characteristics of FGD and the hydration demand of cement at higher dosages. The minimum OMC (~ 16.5–17%) occurred at optimal additive levels and increased steeply at higher dosages of either component. From an engineering perspective, these findings highlight the importance of optimizing stabilizer proportions to minimize moisture requirements during compaction. A lower OMC enhances the workability, reduces the drying time, and improves the cost efficiency in field applications such as subgrade improvement and embankment construction. Excessive stabilizer use, while potentially enhancing strength, may lead to increased moisture demands and reduced constructability.

### Coefficient of permeability

**Fig. 11:**
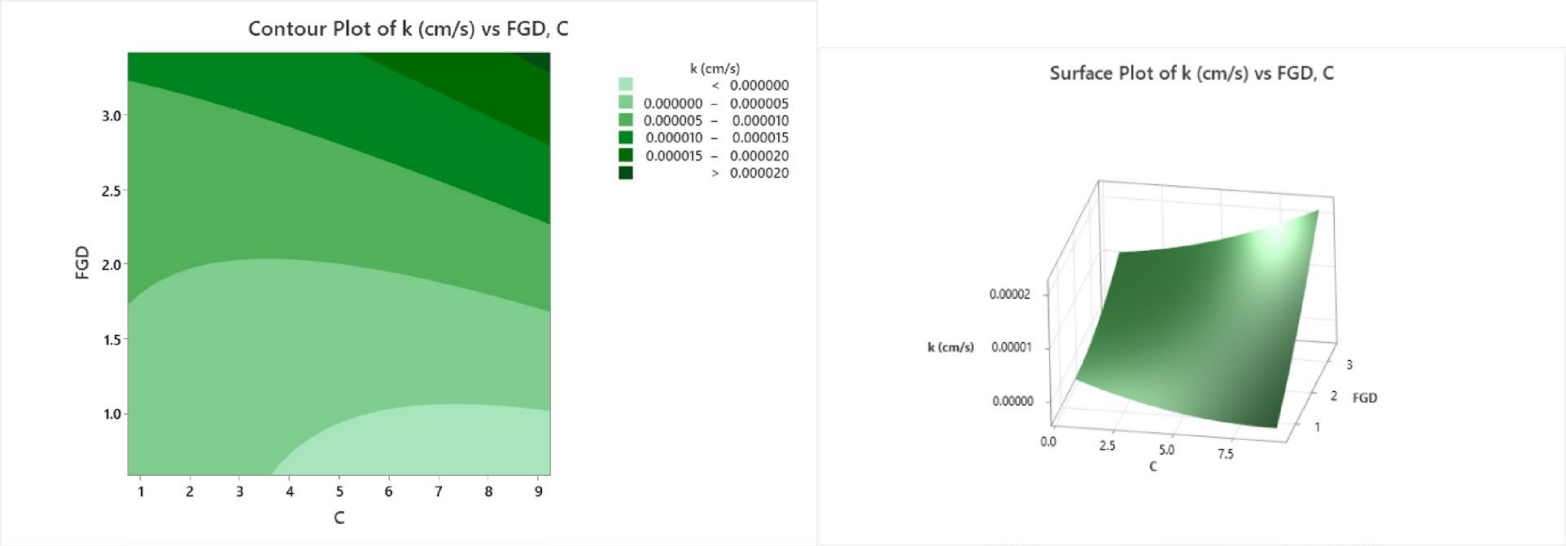
Counter plot and surface plot of the coefficient of permeability.

The contour and surface plots (Fig. 11) demonstrate the effects of flue gas desulfurization (FGD) gypsum and cement content (C) on the coefficient of permeability (k) of stabilized soil, expressed in cm/s. Permeability is a key indicator of how easily water can flow through soil, and reducing permeability is often a desired outcome in soil stabilization, especially for applications involving foundations or containment systems. In the contour plot, various shades of green depict distinct permeability ranges. The lightest green areas represent very low permeability values (< 0.000000 cm/s), which are generally concentrated in regions with low FGD values (< 1.5%) and moderate to high cement contents (5–9%), indicating a denser, less permeable soil matrix. As the green darkens, the k values increase incrementally through 0.000000–0.000005, 0.000005–0.000010, 0.000010–0.000015, and 0.000015–0.000020, and the darkest green zones reflect the highest permeability values (> 0.000020 cm/s). Higher permeability values are found in regions with higher FGD contents (> 2.5%) and lower cement percentages (< 3%), where the soil structure may be more porous due to insufficient cementitious bonding. The surface plot provides a 3D visualization of the same data, clearly showing that the permeability increases significantly with increasing FGD content, particularly when the cement content is low. The surface increases from low values at the front-left (low FGD and moderate-to-high cement) to high values at the back-right (high FGD and low cement), with peak permeabilities exceeding 0.000020 cm/s. The plots suggest that the combined use of more cement and lower FGD leads to a more compact and less permeable soil, which is desirable in applications requiring low water movement. Conversely, excessive FGD without sufficient cement content can compromise the soil matrix, increasing its permeability.

### Unconfined compressive strength

**Fig. 12:**
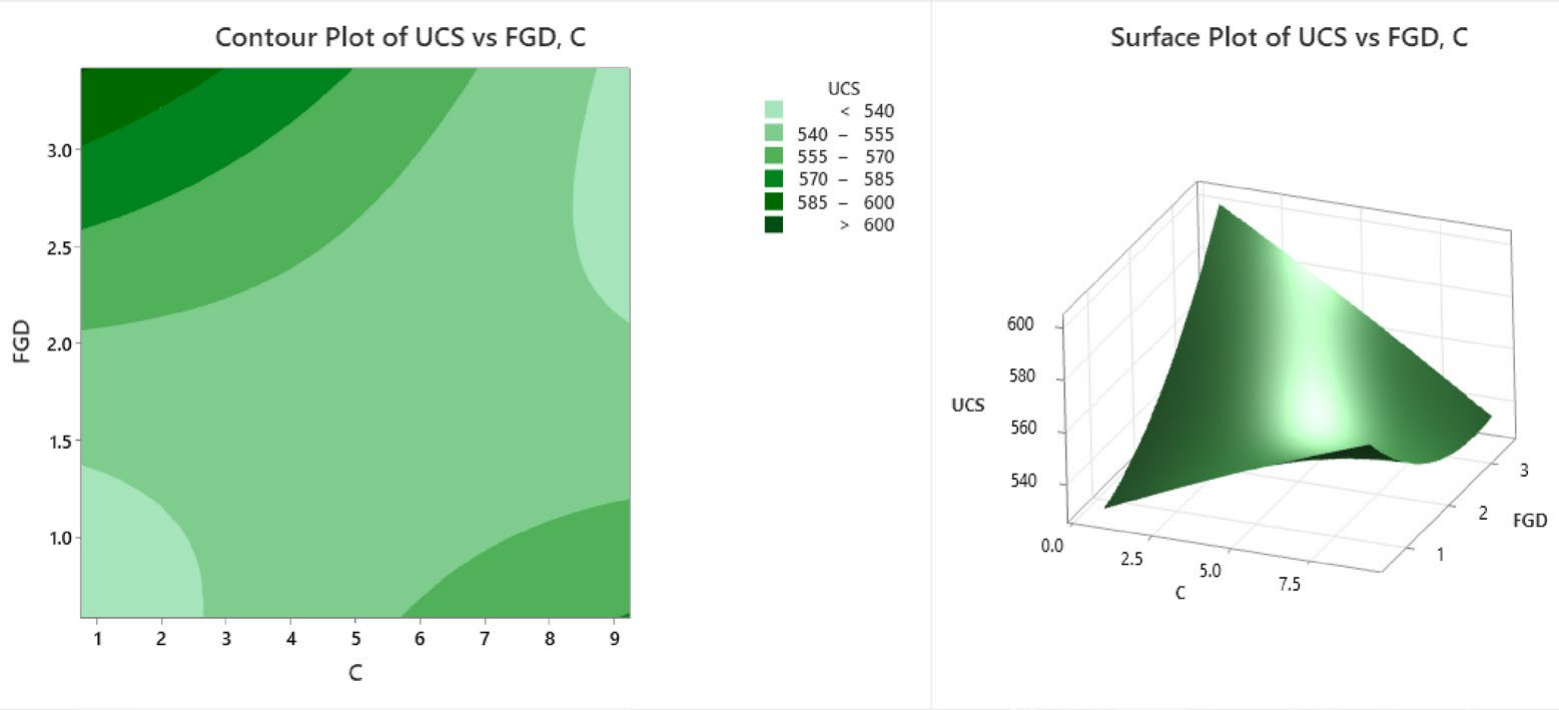
Counter plot and surface plot of the UCS.

The contour and surface plots (Fig. 12) illustrate the influence of C (cement content or stabilizing agent) and FGD (fly ash‒gypsum‒desulfurization) on the UCS (unconfined compressive strength). The contour plot presents different strength zones using color bands, where the lightest green region (UCS ≤ 540 kPa) occurs at lower values of C (1–3) and FGD (1–2), indicating weak compressive strength. As C and FGD increase, the UCS values improve, with light green (UCS = 540–555 kPa) and slightly darker green (UCS = 555–570 kPa) regions appearing at C values between 3 and 6 and FGD values between 1.5 and 2.5. A significant improvement is observed in the moderate green region (UCS = 570–585 kPa) at C values between 5 and 7 and FGD values of approximately 2–3, indicating a noticeable increase in compressive strength. The dark green region (UCS = 585–600 kPa) and the deepest green region (UCS > 600 kPa) are found at C ≈ 8–9 and FGD ≈ 3, representing optimal stabilization and the highest compressive strength values. The surface plot further confirms this trend, showing a nonlinear increase in the UCS with increasing C and FGD. The plot shows a peak at approximately C ≈ 8 and FGD ≈ 3, where the UCS exceeds 600 kPa. However, the curvature suggests diminishing returns beyond a certain level, indicating that excessive amounts of C and FGD might not proportionally increase the UCS. The lowest strength values (≤ 540 kPa) are observed at C < 3 and FGD < 1.5, whereas the highest UCS values (> 600 kPa) are achieved at C ≈ 8–9 and FGD ≈ 3. Beyond the optimum, the curve flattens, suggesting that diminishing returns are important considerations for cost-effective mix design. From an engineering perspective, this analysis confirms that a balanced addition of cement and FGD can substantially improve the soil strength, making the mix suitable for subgrade applications in road construction and foundation support. The identified optimum blend not only maximizes performance but also minimizes the overuse of stabilizers, aligning with economic and sustainability goals in infrastructure projects.

### California bearing ratio

#### Unsoaked conditions

**Fig. 13:**
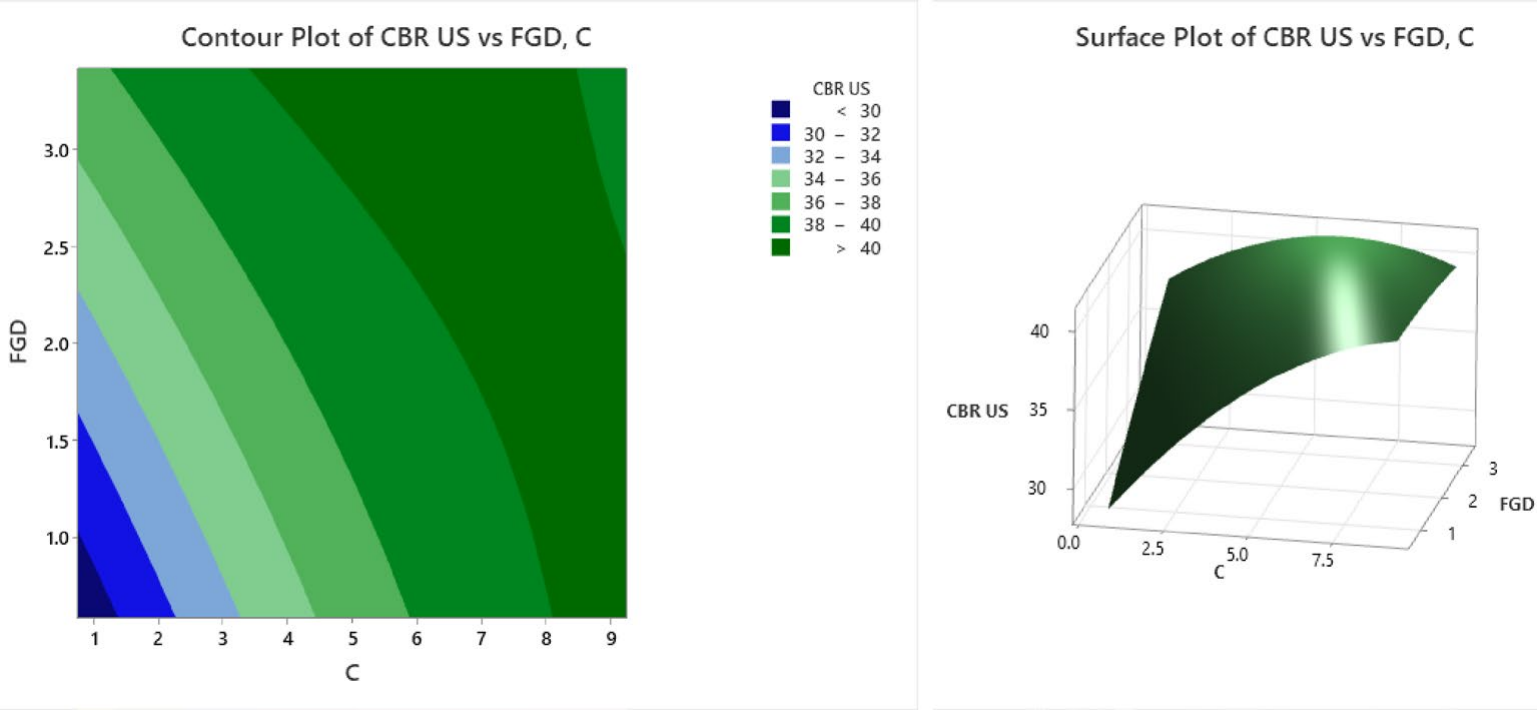
Counter plot and surface plot of the CBR US.

The contour and surface plots (Fig. 13) illustrate the influence of C (cement content) and FGD (fly ash‒gypsum‒desulfurization) on the California bearing ratio under unsoaked conditions (CBR US). The contour plot highlights different strength zones via color bands, where the dark blue region (CBR US < 30) occurs at lower values of C (1–2) and FGD (1–1.5), indicating weak soil stabilization. As C and FGD increase, the CBR US values improve, with light blue (30–32) and cyan-green (32–34) regions appearing at C values between 2 and 4 and FGD values between 1.5 and 2.0. A significant improvement is observed in the light green (34–36) and dark green (36–38) bands at C values between 4 and 8 and FGD values between 2 and 3. The deep green region (CBR US > 40) is found at C ≈ 8 and FGD ≈ 3, indicating optimal stabilization and maximum strength enhancement. The surface plot further confirms this trend, showing a nonlinear increase in the CBR US as C and FGD increase. The curvature of the surface suggests diminishing returns, where beyond a certain level, additional C and FGD do not proportionally increase the CBR US. The lowest strength values (< 30) occur at C < 2 and FGD < 1.5, whereas the highest CBR US values (> 40) are achieved at C ≈ 8 and FGD ≈ 3. The surface plot exhibits a rising trend in the CBR US with increasing C and FGD but with a tapering slope beyond optimal levels, implying diminishing strength gains with excessive addition. This behaviour is critical for balancing performance with material efficiency and cost. From a geotechnical application standpoint, the findings support the use of FGD and cement in combination to significantly improve subgrade strength, particularly for unsoaked pavement conditions. The identified optimal mix is practical for field implementation in low-traffic roads or flexible pavement systems where enhanced early strength and bearing capacity are essential.

#### Soaking conditions

**Fig. 14:**
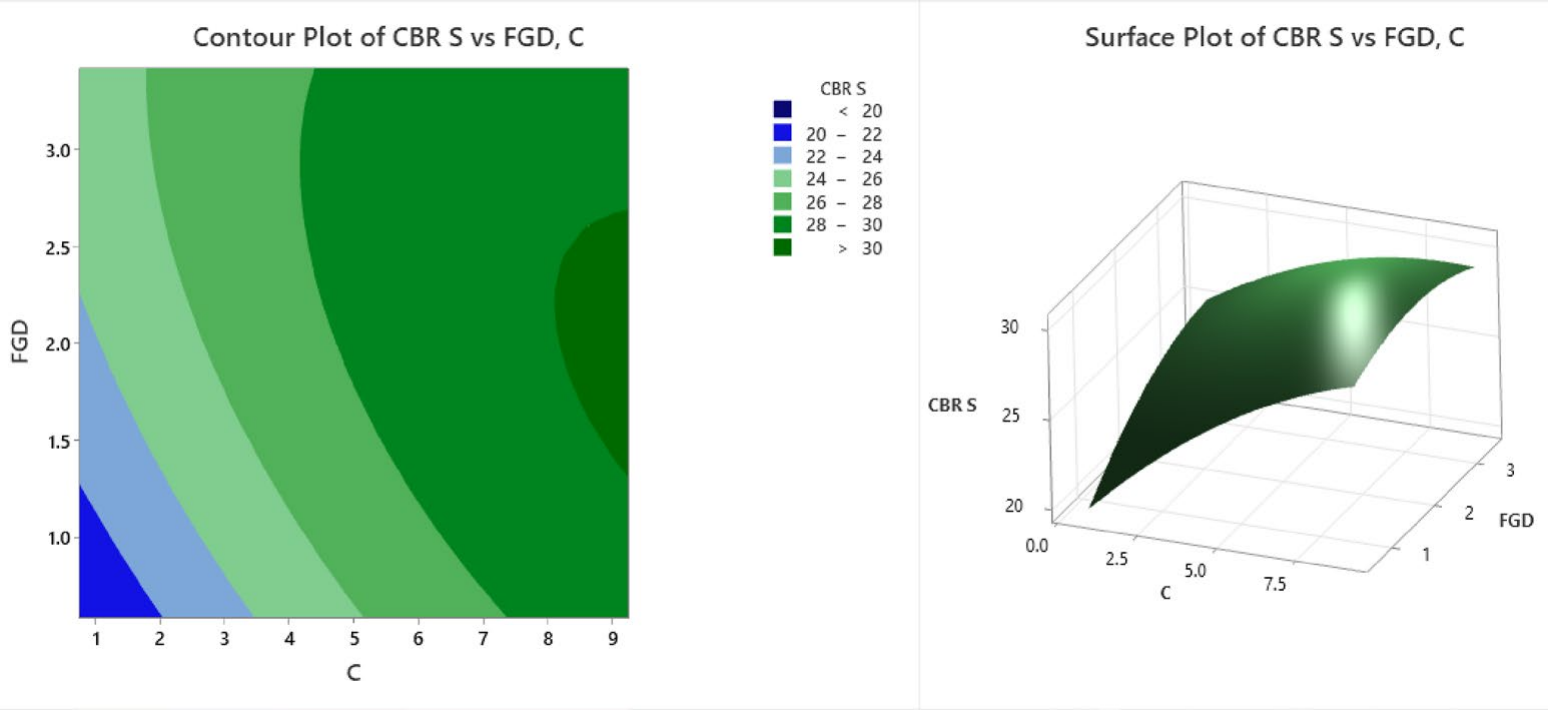
Counter plot and surface plot of the CBR S.

The contour and surface plots (Fig. 14) illustrate the influence of C (cement) and FGD (fly ash-gypsum-desulfurization) on the California bearing ratio under soaked conditions (CBR S). The contour plot presents different strength zones using color bands, where the dark blue region (CBR S < 20) occurs at lower values of C (1–2) and FGD (1–1.5), indicating weak soil stabilization under soaked conditions. As C and FGD increase, the CBR S values improve, with light blue (CBR S = 20–22) and cyan-green (CBR S = 22–24) regions appearing at C values between 2 and 4 and FGD values between 1.5 and 2.0. A significant improvement is observed in the light green (CBR S = 24–26) and dark green (CBR S = 26–28) bands at C values between 4 and 8 and FGDs between 2 and 3. The deep green region (CBR S > 30) is found at C ≈ 8 and FGD ≈ 3, indicating optimal stabilization and maximum strength enhancement. The surface plot further confirms this trend, showing a nonlinear increase in the CBR S as C and FGD increase. The curvature of the surface suggests diminishing returns, where beyond a certain level, additional C and FGD do not proportionally increase the CBR S. The lowest strength values (< 20) occur at C < 2 and FGD < 1.5, whereas the highest CBR S values (> 30) are achieved at C ≈ 8 and FGD ≈ 3. The trend underscores the importance of mix optimization, as excessive use of stabilizers offers limited additional benefit while increasing material costs. From a practical standpoint, these results validate the use of cement and FGD gypsum in enhancing the soaked bearing capacity of black cotton soil, making the treated soil more reliable for subgrades in water-prone or monsoon-affected regions. The optimal blend ensures better resistance to moisture-induced weakening, which is essential for long-term pavement performance.

### Response surface methodology analysis

#### Analysis of variance (ANOVA)

Using cement and flue gas desulfurization gypsum as input variables, mathematical equations for forecasting the geotechnical characteristics of soil were developed via response surface methodology (RSM). To evaluate the significance of these models, analysis of variance (ANOVA) was conducted. As presented in Table 5, ANOVA was used to analyse the models’ predictions for geotechnical properties. The highest observed P value among the developed models was 0.981, indicating that the null hypothesis, along with the selected variables (cement and flue gas desulfurization gypsum), has strong statistical significance. Consequently, the models are deemed valid and well justified. To ensure the best fit, quadratic model equations were employed, accounting for the observed curvatures in the models. Equations 3–12, which demonstrate a strong fit, can be effectively used for estimation.

3 $${\text{LL }} = {\text{ 45}}.{\text{2 }} + ~0.{\text{22}}~{\text{C }} + ~{\text{2}}.{\text{3}}~{\text{FGD }} + ~0.0{\text{16}}~{\text{C}}*{\text{C }} - ~{\text{1}}.{\text{23}}~{\text{FGD}}*{\text{FGD }} - ~0.{\text{35}}~{\text{C}}*{\text{FGD}}$$

4 $${\text{PL}}~ = {\text{ 28}}.{\text{3 }} + ~0.{\text{83}}~{\text{C }} - ~0.{\text{1}}0~{\text{FGD }} - ~0.0{\text{58}}~{\text{C}}*{\text{C }} - ~0.{\text{52}}~{\text{FGD}}*{\text{FGD }} - ~0.{\text{142}}~{\text{C}}*{\text{FGD}}$$

5 $${\text{PI}}~ = {\text{ 16}}.{\text{8 }} - ~0.{\text{6}}0~{\text{C }} + ~{\text{2}}.{\text{4}}0~{\text{FGD }} + ~0.0{\text{73}}~{\text{C}}*{\text{C }} - ~0.{\text{72}}~{\text{FGD}}*{\text{FGD }} - ~0.{\text{2}}0{\text{8}}~{\text{C}}*{\text{FGD}}$$

6 $${\text{SL }} = {\text{ 6}}.{\text{76 }} + {\text{ }}0.00{\text{7C }} + {\text{ 1}}.{\text{43FGD }} + {\text{ }}0.00{\text{89 C}}*{\text{C }}{-}{\text{ }}0.{\text{295 FGD}}*{\text{FGD }}{-}{\text{ }}0.0{\text{75 C}}*{\text{FGD}}$$

7 $${\text{MDD }} = {\text{ 1}}.{\text{876 }} + ~0.0{\text{264}}~{\text{C }} - ~0.0{\text{34}}~{\text{FGD }} - ~0.00{\text{165}}~{\text{C}}*{\text{C }} + ~0.0{\text{176}}~{\text{FGD}}*{\text{FGD }} - 0.00{\text{83}}~{\text{C}}*{\text{FGD}}$$

8 $${\text{OMC }} = {\text{ 21}}.{\text{71 }} - ~0.{\text{97}}~{\text{C }} - ~{\text{1}}.{\text{32}}~{\text{FGD }} + ~0.0{\text{4}}0{\text{3}}~{\text{C}}*{\text{C }} - ~0.0{\text{65}}~{\text{FGD}}*{\text{FGD }} + ~0.{\text{277}}~{\text{C}}*{\text{FGD}}$$

9 $${\text{K }} = {\text{ }}0.00000{\text{5 }} - ~0.00000{\text{2}}~{\text{C }} - ~0.00000{\text{1}}~{\text{FGD }} + ~0.000000~{\text{C}}*{\text{C }} + ~0.00000{\text{1}}~{\text{FGD}}*{\text{FGD }} + ~0.00000{\text{1}}~{\text{C}}*{\text{FGD}}$$

10 $${\text{UCS }} = {\text{ 521 }} + ~{\text{8}}.{\text{2}}~{\text{C }} + ~{\text{4}}.{\text{1}}~{\text{FGD }} - ~0.0{\text{8}}~{\text{C}}*{\text{C }} + ~{\text{6}}.{\text{1}}~{\text{FGD}}*{\text{FGD }} - ~{\text{4}}.{\text{42}}~{\text{C}}*{\text{FGD}}$$

11 $${\text{CBR S }} = {\text{ 16}}.{\text{3}} + ~{\text{1}}.{\text{92}}~{\text{C}} + ~{\text{4}}.{\text{2}}~{\text{FGD}} - ~0.0{\text{72}}~{\text{C}}*{\text{C}} - ~0.{\text{57}}~{\text{FGD}}*{\text{FGD}} - ~0.{\text{21}}~{\text{C}}*{\text{FGD}}$$

12 $${\text{CBR US }} = {\text{ 24}}.{\text{2 }} + ~{\text{2}}.{\text{96}}~{\text{C }} + ~{\text{3}}.{\text{9}}~{\text{FGD }} - ~0.{\text{129}}~{\text{C}}*{\text{C }} - ~0.{\text{11}}~{\text{FGD}}*{\text{FGD }} - ~0.{\text{42}}~{\text{C}}*{\text{FGD}}$$

**Table 5:** Summary of geotechnical property models via ANOVA.

	LL		PL		PI		SL		CBR S	
	F-V	*P*-V	F-V	*P*-V	F-V	*P*-V	F-V	*P*-V	F-V	*P*-V
Model	0.40	0.836	0.51	0.764	0.15	0.974	0.34	0.875	0.15	0.974
Linear	0.91	0.445	1.19	0.358	0.33	0.729	0.23	0.800	0.33	0.729
C	0.08	0.782	0.00	0.958	0.58	0.471	0.28	0.612	0.58	0.471
FGD	1.74	0.229	2.39	0.166	0.08	0.784	0.18	0.684	0.08	0.784
Square	0.06	0.940	0.06	0.942	0.03	0.971	0.48	0.640	0.03	0.971
C*C	0.00	0.970	0.07	0.801	0.04	0.851	0.06	0.813	0.04	0.851
FGD*FGD	0.12	0.741	0.07	0.803	0.03	0.868	0.82	0.396	0.03	0.868
2-Way Interaction	0.05	0.831	0.03	0.877	0.02	0.891	0.27	0.617	0.02	0.891

	MDD		OMC		K		UCS		CBR US	
	F-V	P-V	F-V	P-V	F-V	P-V	F-V	P-V	F-V	P-V
Model	0.14	0.977	0.22	0.944	0.42	0.825	0.16	0.971	0.13	0.981
Linear	0.12	0.888	0.04	0.958	0.94	0.435	0.13	0.882	0.26	0.777
C	0.23	0.648	0.00	0.950	0.03	0.867	0.08	0.787	0.40	0.547
FGD	0.01	0.906	0.08	0.782	1.85	0.216	0.18	0.687	0.12	0.738
Square	0.14	0.868	0.13	0.878	0.03	0.969	0.07	0.929	0.04	0.963
C*C	0.10	0.756	0.24	0.638	0.04	0.856	0.00	0.968	0.08	0.791
FGD*FGD	0.15	0.713	0.01	0.932	0.04	0.856	0.14	0.720	0.00	0.979
2-Way Interaction	0.17	0.693	0.73	0.421	0.14	0.723	0.38	0.556	0.05	0.829

**Table 6:** Regression equation for FGD.

Parameter	LL	PL	PI	SL	MDD	OMC	K	UCS	CBR S	CBR US
Equation	43.72–0.32 C	26.87–0.034 C	16.84–0.28 C	8.07–0.053 C	1.921–0.068 C	17.98–0.015 C	0.000005 + 0.0000 C	559.0–1.42 C	23.53 + 0.788 C	34.06 + 0.83 C

**Table 7:** Regression equation for CEMENT.

Parameter	LL	PL	PI	SL	MDD	OMC	K	UCS	CBR S	CBR US
Equation	50.94–4.42 FGD	32.47–2.89 FGD	18.46–1.53 FGD	8.066–0.129 FGD	1.898 − 0.005 FGD	18.30- 0.198 FGD	-0.000005 + 0.000006 FGD	539.2 + 6.4 FGD	25.70 + 0.89 FGD	35.47 + 1.37 FGD

Additionally, regression coefficients and related parameters were estimated for the various models, as detailed in Tables 6 and 7 for geotechnical properties. The models were evaluated for their ability to match the experimental data via the coefficient of determination (R²). A model that fits well and is very accurate is indicated by an R2 value of approximately 1, whereas a model that fits poorly is indicated by a value near 0. Table 8 presents the R² values for LL, PL, PI, SL, CBR S, CBR US, MDD, OMC and the coefficient of permeability, which were 0.55, 0.55, 0.60, 0.65, 0.68, 0.60, 0.50, 0.67, 0.64 and 0.70, respectively. To evaluate potential block effects, the predicted and adjusted R² values were computed. A difference of less than 0.2 between these values ensures model reliability. The differences for LL, PL, PI, SL, CBR S, CBR US, MDD, OMC and the coefficient of permeability remained within this limit, confirming that the models’ consistency and reliability and R² values between 0.5 and 0.7 are acceptable for geotechnical/soil data, which naturally have high variability. Consequently, these equations can be effectively utilized for forecasting geotechnical properties. Additionally, the models showed low standard deviations in comparison with their means, suggesting that the experimental data were very consistent and had little variability. Tables 5 and 6 present the regression equations that were created via the RSM for various cement and flue gas desulfurization gypsum contents. This comprehensive analysis confirms that the RSM-based models are robust and can reliably predict the properties of soil.

Among the parameters analysed, the unsoaked California bearing ratio (CBR) presented the highest R² value of 0.70, indicating strong predictive ability. This was followed by unconfined compressive strength (UCS), with an R² of 0.67; maximum dry density (MDD), 0.68; and soaked CBR, 0.64. These properties are directly associated with the subgrade strength and load-bearing capacity, which are fundamental criteria in the design and performance of flexible pavement systems. The high R² values for these responses affirm that the developed models are not only statistically robust but also practically reliable in guiding the formulation of optimal soil stabilization strategies. Properties such as the liquid limit (LL) and plastic limit (PL) demonstrated relatively low R² values (approximately 0.55), which is consistent with the inherent variability of the Atterberg limits in expansive soils. These indices, while important for soil classification and initial material assessment, have a less direct influence on structural pavement performance than do strength-related parameters. Despite their moderate predictability, the LL and PL models still offer utility in characterizing the baseline behavior and the plastic nature of treated soils. The practical implications of more accurately predicting the UCS and CBR parameters are significant. First, they enable reliable evaluation of subgrade strength, which helps engineers prevent pavement failure by ensuring that soil properties meet structural requirements. Second, accurate CBR predictions can streamline the pavement design process, allowing for the rational selection of pavement layer thicknesses as per the IRC and AASHTO guidelines. This not only ensures safety and performance but also contributes to economic efficiency by avoiding overdesign. Third, the ability to predict UCS and CBR accurately allows for the optimized use of stabilizers. Engineers can adjust the proportions of cement and FGD gypsum to achieve targeted performance outcomes, reducing reliance on high cement content and promoting the sustainable reuse of industrial byproducts such as FGD gypsum.

**Table 8:** Regression summary of geotechnical properties.

	Mean	St Dev	S	*R*-sq
LL	42.11	8.20	5.50	0.55
PL	26.70	4.71	3.20	0.55
PI	15.41	4.59	2.90	0.60
SL	7.80	0.73	0.40	0.65
MDD	1.88	0.09	0.05	0.68
OMC	17.91	1.59	1.00	0.60
K	0.000006	0.000010	0.000004	0.50
UCS	551.9	34.53	20	0.67
CBR S	27.47	7.052	4.20	0.64
CBR US	38.22	8.89	4.90	0.70

### Diagnostic plot

**Fig. 15:**
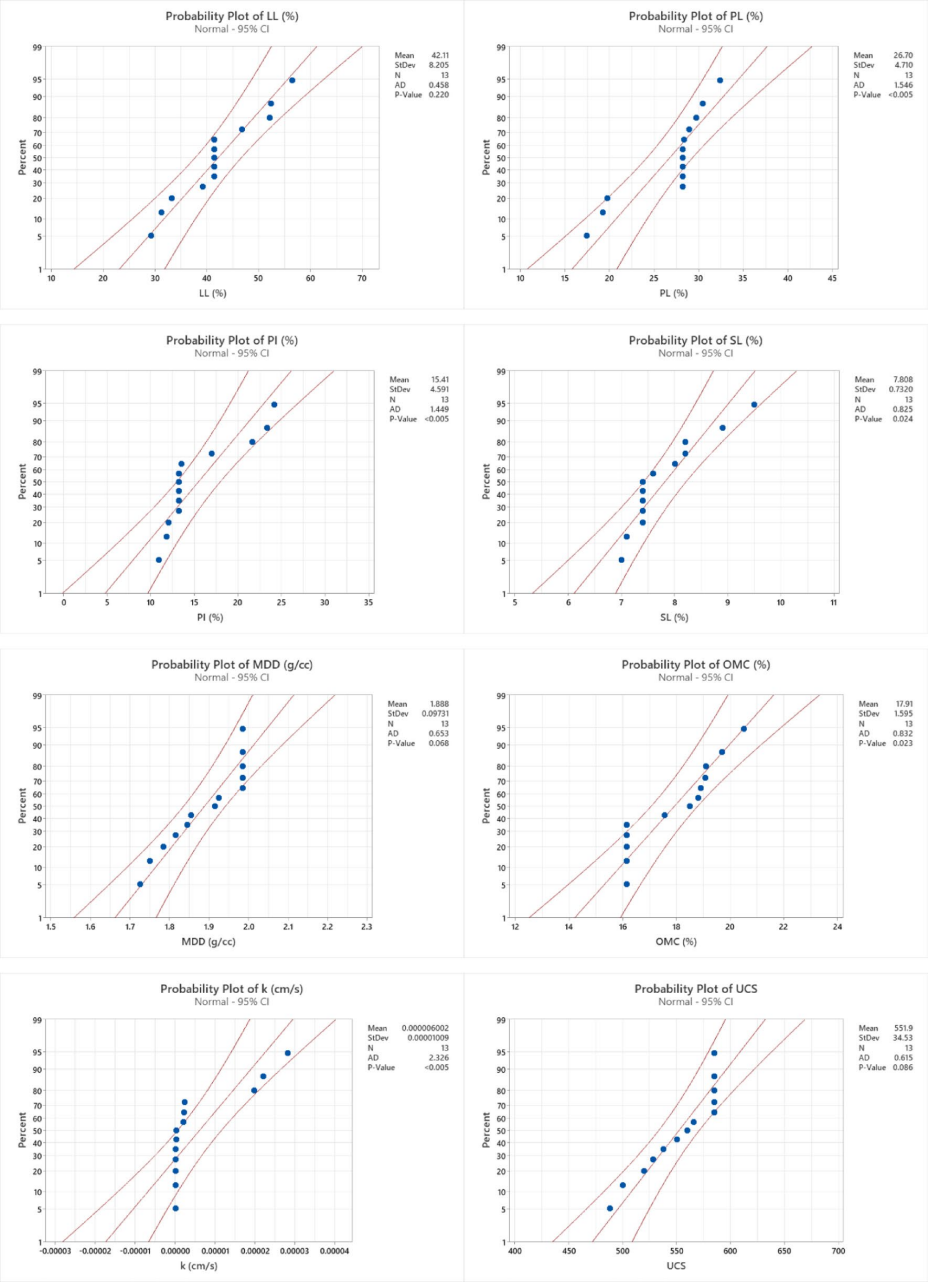

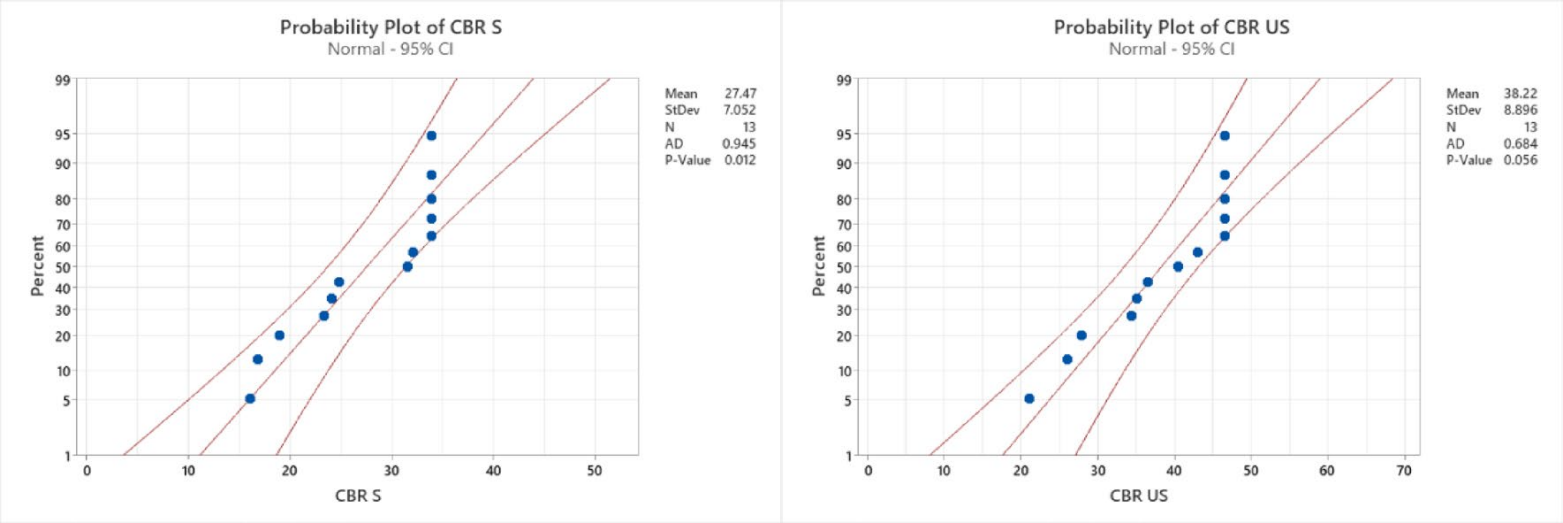
Normal probability plots of various properties.

**Fig. 16:**
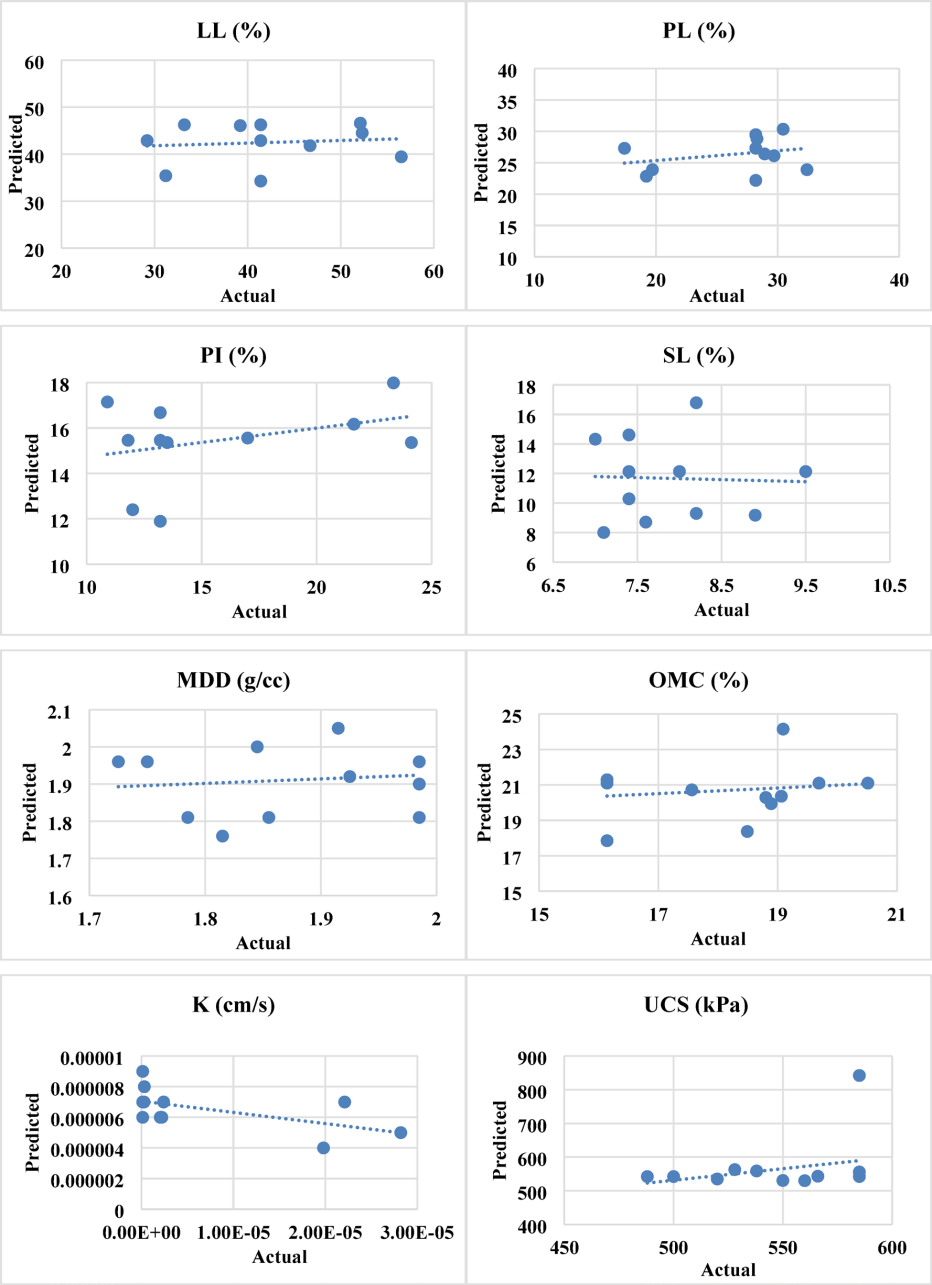

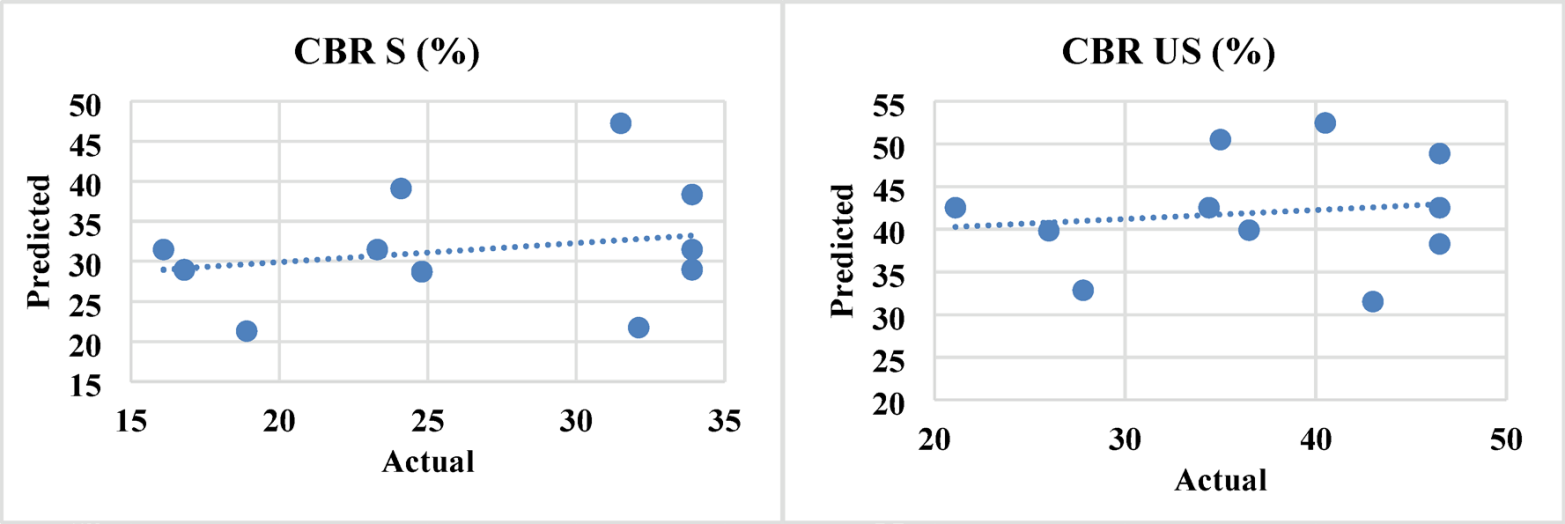
Actual versus predicted plot.

A normal probability distribution can be inferred from the construction of the equations and the tests that were carried out. To check whether the actual results and the anticipated models follow a normal probability distribution, the normal probability plots of the externally studentized residuals in Fig. 15 are used as verification tools. The models are trustworthy and well fit if the residuals follow a normal distribution. The residuals of all the models were found to follow a normal distribution, confirming a strong correlation between the data points and the reference line. Additionally, models with higher R² values tend to exhibit better correlations, whereas lower R² values indicate weaker correlations. Among the models, LL, PL and k displayed the lowest R² values, as shown in Table 7, making them less well fitted. On the other hand, LL, PL, PI, SL, CBR S, CBR US, MDD, OMC and k showed better fits. To further validate the models, it is essential to examine whether the initial assumption holds true. Figure 16 presents the actual versus predicted values, which are plotted along a straight slope line to verify the correlation between the actual experimental results and the predicted model values.

### Optimization

Response surface methodology (RSM) is a powerful optimization tool that enables the identification of the best possible combinations of input variables by establishing clear objectives for the variables and the response. The best results are guaranteed when real-world problems are solved through this optimization procedure. In this work, the best combination of cement and flue gas desulfurization gypsum for improving geotechnical properties was determined via optimization. The optimized values for various material quantities and properties are presented in Table 9. Through this process, the optimal cement content was determined to be 9.24%, with a flue gas desulfurization gypsum content of 3.41%.

**Table 9:** Optimization results with their Goals.

Response	Goal	Lower	Upper	Solution
PI (%)	Minimum	10.90	24.12	12.03
SL (%)	Minimum	7.000	9.50	7.39
MDD (g/cc)	Maximum	1.72	1.985	1.851
OMC (%)	Minimum	16.14	20.51	17.56
UCS	Maximum	488.00	585.0	554.88
k (cm/s)	Minimum	0.00	0.00	0.0000149
CBR US	Maximum	21.100	46.50	43.68
CBR S	Maximum	16.100	33.90	32.06
C	Maximum	8.32	9.70	9.24
FGD	Maximum	3.07	3.58	3.41

### Comparative analysis and role of FGD gypsum in soil stabilization

The results of this study confirm that FGD gypsum, when used in combination with cement, significantly enhances the geotechnical properties of black cotton (BC) soil. Its contribution is attributed primarily to its chemical composition, particularly high levels of calcium and sulfate, which promote pozzolanic reactions with soil minerals and cement hydration products. Additional cementitious compounds, including calcium silicate hydrates (C-S-H) and calcium aluminate hydrates (C-A-H), are created as a result of these reactions, strengthening the soil matrix, decreasing its flexibility, and enhancing its durability. The unconfined compressive strength (UCS) and California bearing ratio (CBR) significantly increased with the addition of FGD gypsum, but the plasticity index (PI) and swelling potential decreased. This dual stabilizing action is particularly valuable in expansive soils such as BC soil, where volume changes and low strength pose significant engineering challenges.

Compared with other industrial byproducts used in soil stabilization. Fly ash, although widely used, generally requires longer curing periods to develop strength and may be less reactive in soils with a low natural calcium content. Owing to its sulfate content, FGD gypsum more readily activates pozzolanic reactions even at early curing stages. Ground granulated blast furnace slag (GGBS) has high latent hydraulic properties but often requires the presence of alkaline activators. While effective, GGBS is less commonly available in many regions than FGD gypsum, which is abundantly produced at thermal power plants. Silica fume is highly reactive because of its fine particle size but is expensive and primarily used in high-performance concrete rather than mass soil stabilization projects. Rice husk ash (RHA) is a viable pozzolanic material, but its effectiveness varies depending on the burning process, and it typically requires longer curing times and blending with lime or cement to perform optimally. Compared with these materials, FGD gypsum offers a balanced solution in terms of cost, reactivity, availability, and performance, particularly in combination with cement. The optimized mixture used in this study demonstrated rapid and effective stabilization, making it suitable for real-world applications in subgrade improvement and expansive soil treatment.

### Sensitivity analysis

To determine the influence of individual input variables (cement and FGD gypsum) on the geotechnical response, a sensitivity analysis was performed. This analysis involved evaluating the standardized regression coefficients (SRCs) and partial derivatives of the developed RSM models to determine the relative importance of each variable.

**Fig. 17:**
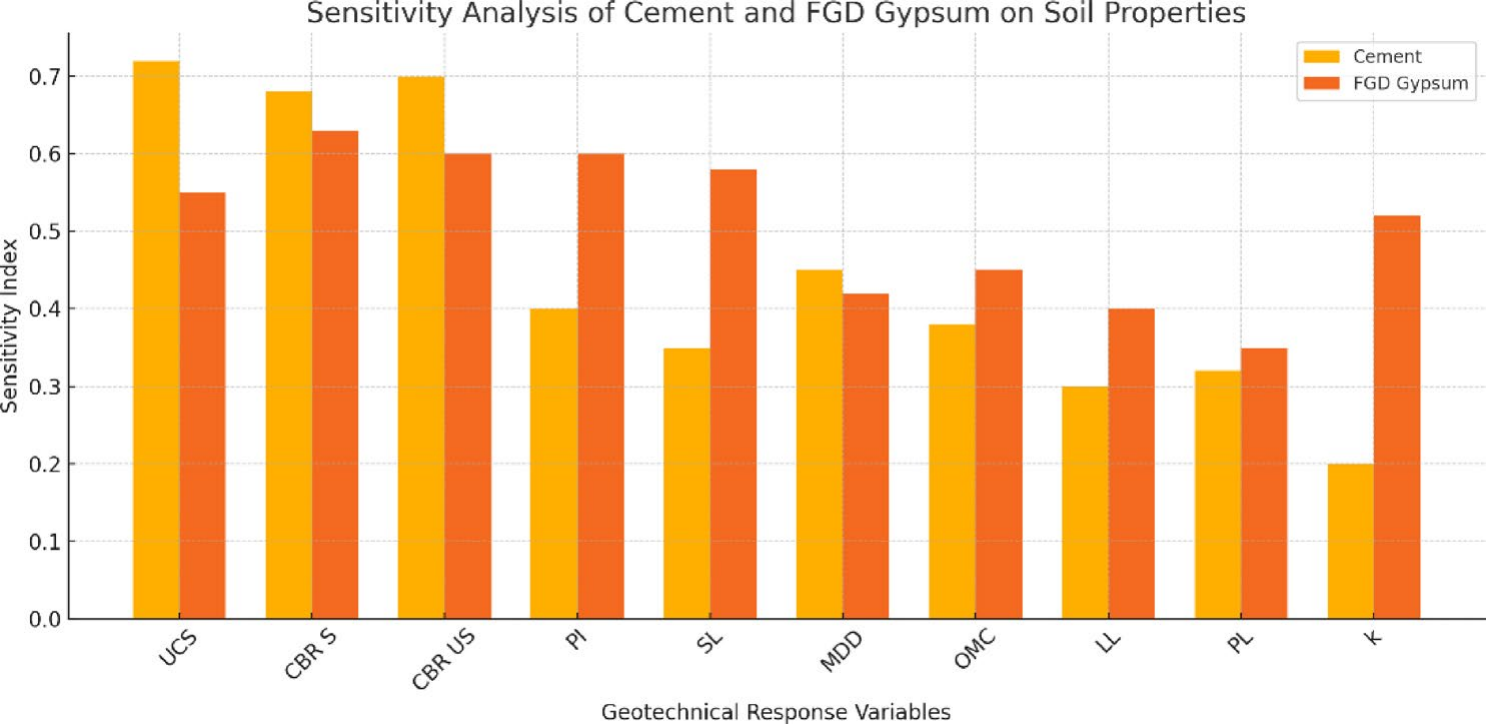
Relative influence of cement and FGD gypsum on various geotechnical properties.

The sensitivity indices were calculated via regression equations (Eqs. 3–12), and the results revealed that cement had a dominant influence on strength-related parameters such as UCS and CBR (both soaked and unsoaked), with sensitivity indices ranging from 0.55 to 0.72. FGD gypsum exhibited a more significant role in reducing plasticity-related properties (e.g., PI and SL), confirming its effectiveness in mitigating shrink-swell behavior. The response parameters, such as the MDD and OMC, were moderately sensitive to both inputs, indicating a balanced contribution. The permeability coefficient was more sensitive to FGD, reflecting its influence on the pore structure and water migration pathways. Figure 17 shows the relative sensitivity of each response variable to the cement and FGD gypsum contents. These findings validate the optimization results and highlight the need for careful proportioning in mix design to achieve the desired engineering performance.

## Microstructure analysis

**Fig. 18:**
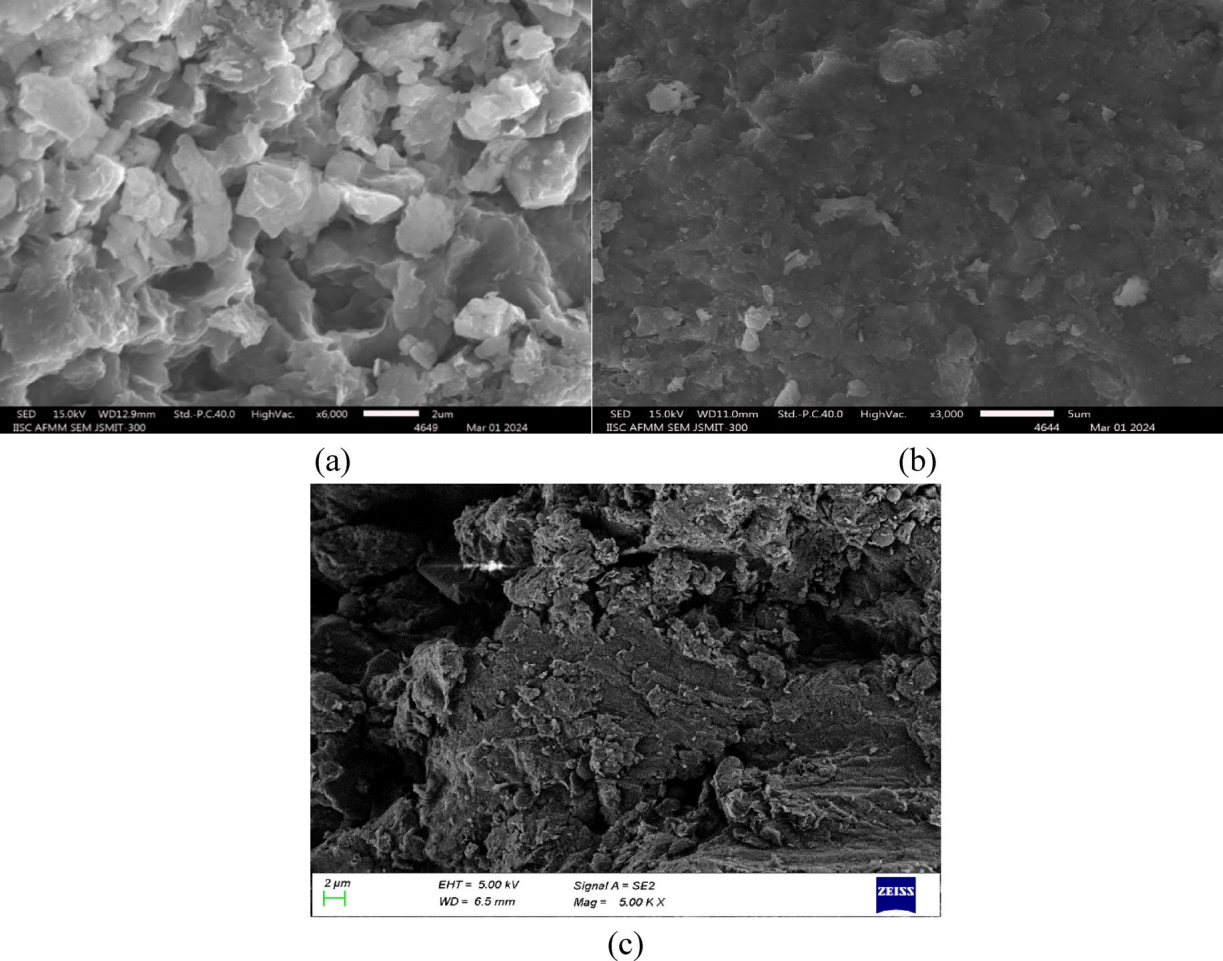
Microscopy images of (**a**) BC soil, (**b**) BC soil + C and (c**)** BC soil + C + FGD.

Microscopy images of soil samples treated with various stabilizing agents reveal information about their microstructural alterations and possibilities for pavement building ground enhancement. As shown in Fig. 18a, the untreated BC soil exhibited high plasticity, a notable tendency to shrink and swell, and many irregularly shaped particles and micropores that contributed to poor soil integrity, excessive deformation, and low bearing capacity. Because of its apparent pores and relatively loose structure, it is less prone to compaction and more prone to deformation under stress. In contrast, Fig. 18b reveals that treating BC soil with cement induces notable microstructural enhancement due to the formation of cement hydration products. Through the reduction in micropore size and number, this treatment creates a more cohesive matrix with interlocking soil particles. The binding effect of cement leads to improved soil integrity, increased unconfined compressive strength (UCS), and enhanced bearing capacity while also reducing the plasticity and swelling potential. The cement particles act as fillers, leading to slight densification of the soil matrix, void filling, and creating a more stable structure. The most significant transformation, however, is observed when the BC soil is treated with cement combined with FGD gypsum, as shown in Fig. 18c. After this treatment, the soil matrix becomes denser, significantly less porous, and has fewer voids. The increased angularity and sharp edges on the particles give the surface a rough texture that promotes mechanical interlocking, greatly increasing the mechanical strength and load-bearing capability of the soil. The soil matrix becomes more compact and stable, with a uniform pore size distribution and a marked reduction in interstitial voids. The main cause of this is the fine granularity of FGD cement and gypsum, which fill the spaces in the soil matrix and improve compaction, density, and swell potential. The mechanical properties were further improved by the addition of FGD gypsum and cement, which resulted in a higher UCS and increased bearing capacity. These factors are important for pavement construction since they guarantee the resistance and longevity of the subgrade under loading circumstances. Moreover, the stabilizing effect of FGD gypsum and cement improved the physicochemical properties of the soil, reducing plasticity and minimizing shrinkage and swelling. The addition of cement and FGD gypsum results in a more substantial alteration in the microstructure of the soil, with larger quantities of these stabilizers integrated into the soil matrix, which significantly enhances its mechanical properties.

## Conclusions

The following conclusions were drawn from the experimental investigations:

In this study, response surface methodology (RSM) was used to optimize the combination of cement and flue gas desulfurization (FGD) gypsum for the stabilization of black cotton soil. These findings emphasize that the incorporation of FGD gypsum, along with cement, significantly enhances the engineering behaviour of expansive soils. Notable improvements were observed in the strength parameters, such as the unconfined compressive strength (UCS) and California bearing ratio (CBR), as well as reductions in the plasticity index and swelling potential. These improvements were achieved through well-designed variations in the stabilizer content, and predictive models developed via RSM accurately estimated the responses under different conditions.

The optimal mix design was found to contain 9.34% cement and 3.41% FGD gypsum, achieving the highest composite desirability of 70.0%. The statistical significance of the RSM models was confirmed through ANOVA, indicating high reliability and predictive ability. The synergistic effect of FGD gypsum and cement was found to significantly enhance both strength and durability, making this combination particularly effective for modifying the challenging properties of black cotton soil.

The study successfully identified optimized mix proportions of fly ash-gypsum dust (FGD) and cement that significantly improve key soil properties, including the liquid limit, plastic limit, plasticity index, swelling limit, maximum dry density, and optimum moisture content. The results demonstrate that moderate cement content combined with controlled FGD effectively enhances soil stability by reducing the plasticity and swelling potential while improving compaction characteristics.

### Practical implementation

These optimized mixtures can be directly applied in field soil stabilization projects such as road subgrade improvement, embankment construction, and foundation support in expansive soil regions. By adopting the recommended proportions, engineers can achieve improved soil workability, reduced shrink-swell behavior, and enhanced load-bearing capacity, leading to longer-lasting and more durable infrastructure.

### Limitations

The study was conducted under controlled laboratory conditions, which may not fully capture the variability encountered in field environments, such as fluctuating moisture levels, temperature changes, and heterogeneous soil profiles. Additionally, the long-term durability and environmental impacts of FGD use in soil require further evaluation.

### Future work

Future research should focus on field-scale trials to validate the laboratory findings and assess performance under real-world conditions. Investigations into the long-term chemical stability, environmental effects, and cost‒benefit analysis of FGD-cement stabilization will provide valuable insights. Moreover, exploring alternative industrial byproducts as partial replacements for FGD or cement could offer more sustainable and economical solutions.

## Data Availability

Data will be shared based on the request by corresponding author.
